# Multiscale modelling of birth–death processes

**DOI:** 10.1007/s00285-026-02448-5

**Published:** 2026-08-03

**Authors:** Tom Kimpson, Domenic P. J. Germano, Jennifer A. Flegg, Mark B. Flegg

**Affiliations:** 1 School of Mathematics and Statistics, The University of Melbourne, Parkville, VIC 3010 Australia; 2 ARC Centre of Excellence for the Mathematical Analysis of Cellular Systems (MACSYS), The University of Melbourne, Parkville, VIC 3010 Australia; 3 School of Mathematics, Monash University, Clayton, VIC 3800 Australia

**Keywords:** Hybrid simulation, Birth–death, JSF, PDMP, Extinction, Lotka–Volterra, 92D25, 60J80, 60J28, 60J25, 65C05

## Abstract

Many biological systems exhibit multiscale dynamics, where some species occur in high copy numbers while others remain rare. This heterogeneity necessitates hybrid modelling approaches: deterministic models are computationally efficient but inaccurate for low-count species, while fully stochastic simulations are accurate but prohibitively expensive. Threshold-based hybrid methods, such as the Jump–Switch–Flow (JSF) algorithm, address this by simulating low-count species stochastically and high-count species deterministically, switching at a user-chosen threshold Ω. In such methods, the choice of Ω controls the trade-off between computational cost and accuracy, but is typically made by trial-and-error: there is no principled way to choose Ω *a priori* for a given observable of interest. We close this gap for extinction probability. Our contribution is a computable, method-agnostic error bound that quantifies the discrepancy introduced by the threshold and yields an explicit rule for selecting Ω to meet a user-specified error tolerance. We formalise JSF as a piecewise-deterministic Markov process and derive backward equations for extinction under exact and hybrid dynamics. Near extinction boundaries, the complex nonlinear dynamics reduce to tractable time-inhomogeneous linear birth–death processes; this structure yields a rigorous error decomposition into early and late excursions, whose dominant term becomes a fast, actionable heuristic requiring only the solution of a scalar Riccati equation. Monte Carlo studies on a stochastic Lotka–Volterra model confirm that the heuristic reliably upper-bounds the empirical error in extinction probability across a wide parameter range. The framework depends only on the birth and death rates near extinction, not on the specific simulation method, and therefore applies beyond JSF to any threshold-based hybrid simulation scheme.

**Supplementary Information:**

The online version contains supplementary material available at 10.1007/s00285-026-02448-5.

## Introduction

Population dynamics in biological systems frequently navigate vast orders of magnitude across temporal and spatial scales. In epidemiology, infectious diseases emerge from molecular interactions within individual hosts, spread through local transmission networks, and can ultimately manifest as global pandemics affecting millions (Childs et al. 2019; Doran et al. 2023; Wang et al. 2022). In ecology, populations demonstrate dramatic “boom-and-bust” cycles where abundances fluctuate across orders of magnitude (Ludwig et al. 1978; Levin 1992; Nichols et al. 2008; Mcgarigal et al. 2016). In systems biology, cellular and molecular processes cascade through tissue-level organization to produce whole-organism phenotypes (Dada and Mendes 2011; Walpole et al. 2013; Qu et al. 2011).

At the finest scale, discrete-state, continuous-time Markov chains (CTMCs) provide the most physically accurate description of spatially well mixed biological systems, explicitly modelling the probabilistic nature of individual events. The foundational method for simulating CTMCs is the Doob-Gillespie Stochastic Simulation Algorithm (SSA) (Doob 1942, 1945; Gillespie 1976, 1977), which serves as the “gold standard” for accuracy by capturing the true stochastic dynamics of discrete biological processes. However, the SSA’s computational cost presents a significant barrier to practical application. The algorithm simulates every single event, making it prohibitively computationally expensive for systems involving large populations or rapid reaction rates, rendering it impractical for most realistic biological systems.

To address these computational limitations while retaining stochasticity, several fast-but-approximate methods have been developed, such as the tau-leaping algorithm (Gillespie 2001) and the chemical Langevin equation (Gillespie 2000; Rao and Arkin 2003; Cao et al. 2006; Gibson and Bruck 2000; Simoni et al. 2019). These approaches accelerate simulation by approximating multiple reaction events collectively or by treating fluctuations as continuous noise. However, while they can be effective in certain regimes, the error incurred by these approximations is often unacceptable when modelling systems where rare events, absorption states, or precise stochastic dynamics play a critical role.

At larger scales, continuous deterministic models such as ordinary differential equations (ODEs) provide an efficient alternative. ODEs are computationally efficient and effective for systems with large numbers of interacting components (such as vast populations of cells, molecules, or organisms) because at such large scales, random fluctuations in individual events (births, deaths, reactions) tend to average out, justifying the continuum approach. Indeed, many ODEs used in biology can be mathematically justified as the “ensemble average” behaviour of an underlying stochastic process in the limit of large populations (Kurtz 1970, 1971, 1972). However, this continuum assumption comes with significant drawbacks. At smaller scales, the inherent stochasticity of individual events can no longer be averaged out, and the continuous ODE treatment of discrete processes can lead to artefacts such as “atto-fox problems” where populations shrink to unphysically small sizes that persist indefinitely, whereas in reality the last individual would have died out (Fowler 2021; Lobry and Sari 2015). Accordingly, ODE models are incapable of meaningfully addressing threshold-sensitive or stochasticity-driven phenomena. A prime example is absorption into extinction states which are ubiquitous in biological systems yet fundamentally require stochastic treatment.

When the system dynamics remains firmly in a single large-scale or small-scale regime, the choice between continuous or discrete modelling approaches is clear. However, for multiscale systems that traverse both large and small population regimes, neither approach is sufficient on its own. This fundamental gap creates the central need for hybrid methodologies, as deterministic models are inaccurate for small populations and stochastic models become computationally infeasible for large ones. Developing methods that can faithfully bridge these scales has been a long-standing goal in applied mathematics (Cotter and Erban 2016; Flegg et al. 2014; Isaacson 2013).

To bridge the gap between deterministic and stochastic approaches, a variety of hybrid modelling methods have been developed. These methods aim to provide a “best of both worlds” solution by dynamically partitioning a system and applying the most appropriate simulation technique to each partition, thereby combining the accuracy of stochastic models for small populations with the speed of deterministic models for large ones. Classic approaches include partitioned hybrid schemes which approximates fast reactions deterministically while simulating slow reactions stochastically (Haseltine and Rawlings 2002), and partitioned leaping methods that categorise reactions as fast or slow (Cao et al. 2005). A parallel line of work partitions by *species abundance* using population thresholds, typically augmented with a middle Chemical Langevin Equation (CLE) regime between the SSA and the deterministic ODE, as in the multi-level schemes of Winkelmann and Schütte (2017, 2020); see Winkelmann and Schütte (2020) for a comprehensive review. Further innovations include the regime-conversion method (Kynaston et al. 2023); jump–diffusion processes (Buckwar and Riedler 2011; Angius et al. 2015); piecewise deterministic Markov processes (PDMPs) (Alfonsi et al. 2004; Marchetti et al. 2016; Crudu et al. 2009); and the “Jump-Switch-Flow” method (Germano et al. 2024). For a broader review of hybrid multiscale modelling approaches, we refer the interested reader to Simoni et al. (2019).

A central unresolved question in applying threshold-based hybrid methods is how to choose the threshold parameter Ω *a priori* so that the error in a chosen observable can be controlled against a target tolerance. For example, in Germano et al. (2024), Ω was set heuristically by running trial simulations and selecting a value that resulted in accurate, but reasonable, computational performance. In this work we provide a systematic method for selecting Ω *a priori*, without the need for extensive exploratory runs. We note that the “optimal” choice of Ω is not universal: it depends on the biological or epidemiological question under investigation, as well as the willingness to sacrifice accuracy for computational efficiency. For example, if the research question concerns accurately quantifying the probabilities of extinction or the timing of rare stochastic events, a relatively low Ω is often sufficient. This choice maximises computational efficiency by using the deterministic model at high counts, while critically retaining the essential stochastic dynamics as the population approaches the extinction boundaries. Conversely, if the primary aim is to capture complex dynamics like stochastic cycles, or if the mean-field model is known to be a poor approximation even at high population sizes, a larger Ω may be necessary. This ensures that stochastic dynamics are retained over a much broader range of the state space, at the cost of computational speed.

In this study, we use the Jump-Switch-Flow (JSF) hybrid method as our simulator, and focus exclusively on the objective of quantifying extinction probabilities accurately within a finite time horizon. We develop a systematic method for selecting a regime switching threshold, Ω, that balances computational efficiency and accuracy: we seek the smallest threshold that keeps the error between the exact SSA solution and the JSF hybrid solution within a prescribed tolerance. Specifically, we derive a method to calculate an upper bound on the error in extinction probability introduced by the hybrid approximation. We focus on extinction events due to their ubiquitous biological and epidemiological significance. Extinction events, including viral clearance, species extinction, and the eradication of invasive populations, are fundamentally predicated on low-population stochasticity. This makes extinction an ideal case study to demonstrate how a principled selection of Ω can be tailored to a specific scientific objective.

Our analytical approach exploits a key simplification: near extinction boundaries, the complex nonlinear dynamics of realistic biological systems reduce to tractable birth–death processes. When populations are small, interactions between rare individuals become negligible, and each individual’s fate can be analysed independently. This birth–death structure enables exact computation of extinction probabilities via classical branching process theory, and provides the mathematical foundation for our error analysis.

This paper is organised as follows. Section 2 introduces the three modelling frameworks: deterministic ODEs, stochastic CTMCs as the gold standard of accuracy, and JSF hybrid methods. Section 3 develops the mathematical framework underpinning our analysis, showing how general systems reduce to birth–death processes near extinction; this reduction is the key that enables the error bounds derived in Sect. 4. Although we work with JSF throughout for concreteness, the derivation depends only on the birth and death rates of the scarce species near the extinction boundary, so the resulting bounds apply to any threshold-based hybrid scheme; we return to this point in Sect. 6. Building on this foundation, Sect. 4 derives theoretical error bounds and practical heuristics for hybrid threshold selection. Section 5.1 demonstrates the theory through a detailed case study using the stochastic Lotka-Volterra predator–prey system, with explicit solutions and numerical validation. Lastly, we discuss the broader implications of the work and future extensions in Sect. 6.

## Modelling frameworks

This section outlines the three mathematical frameworks used to model population dynamics: the exact stochastic description, the deterministic large-population limit, and a hybrid method that combines elements of both.

### The stochastic model: continuous-time Markov chains (CTMC)

The most faithful representation of stochastic events occurring in a well-mixed population system is by a Continuous-Time Markov Chain (CTMC). Let the state of the system at time t≥0 be the vector of species populations, $\textbf{X}(t)=(X_1(t),\dots ,X_N(t)) \in \mathbb {N}_0^N$. The system evolves through a set of *R* possible reactions. Each reaction *r* is defined by:

- A *stoichiometric vector* $\boldsymbol{\nu }_r \in \mathbb {Z}^N$, which describes the change in population counts when reaction *r* occurs.
- A *propensity function* $a_r(\textbf{x},t) \ge 0$, which gives the instantaneous probability rate of reaction *r* occurring, given the system is in state $\textbf{x}$ at time *t*.

The dynamics of the CTMC are fully captured by its generator, $\mathcal {L}_t$. Acting on a bounded test function $f:\mathbb {N}_0^N \rightarrow \mathbb {R}$, the generator describes the instantaneous expected rate of change of $f(\textbf{X}(t))$ (Anderson and Kurtz 2015)

1 $$\begin{aligned} (\mathcal {L}_t f)(\textbf{x}) = \sum _{r=1}^R a_r(\textbf{x},t) \big [f(\textbf{x}+\boldsymbol{\nu }_r)-f(\textbf{x})\big ]. \end{aligned}$$

The generator is related, by duality, to the forward (Kolmogorov) operator ($\mathcal {L}_t^*$), which acts on the probability distribution $(p(\textbf{x},t) = \Pr [\textbf{X}(t)=\textbf{x}])$

2 $$\begin{aligned} \frac{d}{dt} p(\textbf{x},t) = (\mathcal {L}_t^*p)(\textbf{x},t)= \sum _{r=1}^R \big [a_r(\textbf{x}-\boldsymbol{\nu }_r,t)p(\textbf{x}-\boldsymbol{\nu }_r,t) - a_r(\textbf{x},t)p(\textbf{x},t)\big ], \end{aligned}$$

with the generator and its adjoint satisfying

3 $$\begin{aligned} \frac{d}{dt}\mathbb {E}[f(\textbf{X}(t))] = \mathbb {E}[(\mathcal {L}_t f)(\textbf{X}(t))]. \end{aligned}$$

Events such as extinction are handled by defining an absorbing set $\mathcal {A} \subseteq \mathbb {N}_0^N$, where the process stops once it enters. While this approach provides our most accurate description of stochastic population dynamics, simulating every individual event becomes computationally prohibitive for large populations.

### The deterministic limit: mean-field ODEs

In systems where all population counts are very large, stochastic effects become negligible. In this regime, the law of large numbers guarantees that the system’s behaviour converges to a deterministic path. This path is described by a set of Ordinary Differential Equations (ODEs), often called the mean-field or Kurtz limit:

4 $$\begin{aligned} \dot{\textbf{x}}(t) = \sum _{r=1}^R \boldsymbol{\nu }_r \, a_r\big (\textbf{x}(t),t\big ), \qquad \text {with initial condition} \quad \textbf{x}(0)=\textbf{x}_0 \in \mathbb {R}_{\ge 0}^N. \end{aligned}$$

While computationally efficient, with standard numerical methods such as Euler and Runge–Kutta readily available, this model completely ignores the random fluctuations that are critical for describing phenomena like extinction in low-population systems. Despite the name, mean-field ODE models do not necessarily describe the mean behaviour of the CTMC; in fact, for non-linear systems, they rarely do.

### The Hybrid model: jump-switch-flow (JSF)

Neither the CTMC nor ODE approach alone are sufficient for describing multiscale biological systems. The core challenge is that these systems often exhibit a mixture of reactions occurring on vastly different timescales (i.e., ‘fast’ and ‘slow’ reactions simultaneously). This ‘stiffness’ problem is then further compounded by populations that can transition between high-count (continuum) and low-count (discrete) regimes. The JSF process (Germano et al. 2024) addresses these challenges by dynamically blending the accuracy of the CTMC at low populations with the efficiency of the ODE model at large populations. We adopt JSF here for two reasons: it retains a discrete, integer-valued population count so that true extinction is modelled exactly (avoiding the “atto-fox” problem of ODEs and the negative-population pathology of some tau-leaping schemes) while remaining fast enough to support computationally intensive workflows such as Bayesian parameter inference. A user-friendly public implementation is available.^1^ The core idea is to partition the *N* species compartments dynamically based on a vector of integer thresholds, $\boldsymbol{\Omega }=(\Omega _1,\dots ,\Omega _N) \in \mathbb {N}^N$. At any time *t*, we define two index sets:

5 $$\begin{aligned} J(t)=\{i: X_i(t) \le \Omega _i\} \quad \text {and} \quad F(t)=\{1,\dots ,N\} \setminus J(t). \end{aligned}$$

The species with indices in *J*(*t*) are treated as stochastic, while those with indices in *F*(*t*) are treated as deterministic.

The temporal evolution proceeds as follows:

- *Jump:* Discrete stochastic events affect the components $\textbf{X}_J$ (simulated exactly using algorithms like the Stochastic Simulation Algorithm), and also impact species in *F* through reactions involving *J*. Propensities are calculated using the full hybrid state $(\textbf{X}_J, \textbf{x}_F)$. Importantly, the propensity usually depends continuously on time due to changing populations in *F*. This is addressed using an event ‘clock’ system (Germano et al. 2024).
- *Switch:* Whenever any component crosses its threshold $\Omega _i$ in either direction, the partition sets *J*(*t*) and *F*(*t*) are instantaneously recomputed, and the governing dynamics for that species immediately switch between stochastic and deterministic treatment.
- *Flow:* Between discrete events, the deterministic components $\textbf{x}_F$ evolve continuously according to the ODE system Eq. (4), restricted to the species with indices in *F*(*t*), and reactions involving only those species. Since $\textbf{X}_J$ remains constant between jumps and is small by definition, its influence on the flow dynamics can often be neglected.

This process is a type of Piecewise-Deterministic Markov Process (PDMP Davis 1984). The dynamics between any two switching events (i.e. for a fixed partition *J*(*t*) and *F*(*t*)) are described by the hybrid generator, $\mathcal {G}_t$. This generator generalises the CTMC generator $\mathcal {L}_t$ by combining discrete jumps with continuous flow. Denoting the combined hybrid state by $\textbf{z}:= (\textbf{X}_J, \textbf{x}_F)$, for a function *f* on the hybrid state space:

6 $$\begin{aligned} (\mathcal {G}_t f)(\textbf{z}) = \sum _{r\in \mathcal {R}_J} a_r(\textbf{z},t)\big [f(\textbf{z}+\boldsymbol{\nu }_r)-f(\textbf{z})\big ] + \nabla _{\textbf{x}_F} f \cdot \sum _{r\in \mathcal {R}_F} \boldsymbol{\nu }_r\, a_r(\textbf{z},t), \end{aligned}$$

where the first term captures stochastic jumps and the second term captures deterministic flow. The reaction sets $\mathcal {R}_J$ and $\mathcal {R}_F$ partition the reactions based on which components they affect (for a detailed treatment of this partitioning, see (Germano et al. 2024)).

## Birth–death framework for extinction analysis

Before deriving error bounds, we establish the mathematical framework. The JSF method aims to preserve key properties of the exact CTMC. Which properties matter depends on the scientific question under investigation: for instance, one might ask *what is the probability of extinction within a given time horizon?* or *what is the expected species occupancy?* The choice of question defines the objective against which the threshold Ω should be optimised. Henceforth, we consider a common switching threshold across species and write simply Ω (i.e., $\Omega _i = \Omega$ for all *i*). If Ω is set sufficiently large, then the JSF method approaches the CTMC, preserving accuracy but sacrificing computational efficiency. Conversely, while we desire a suitably small Ω to minimise computational expense, setting Ω too small risks introducing unacceptable error in the property of interest.

In this work, we focus on the first of these questions: the probability of species extinction within a finite time horizon. We use this as our metric to evaluate the JSF hybrid method’s accuracy. The choice is both biologically meaningful and mathematically tractable; extinction events are quintessentially discrete phenomena and cannot be described by continuous models. Our analytical strategy exploits the birth–death structure that emerges near extinction boundaries. When populations are small, the complex non-linear dynamics of the full system reduce to simpler birth–death processes. Specifically, we show how a general continuous-time Markov chain (CTMC) model of a reaction network reduces to a time-inhomogeneous linear birth–death process near extinction, where a subset of species is scarce. This birth–death framework offers three key advantages. First, it provides exact discrete representation of extinction events. Second, extinction probabilities can be calculated analytically via backward Kolmogorov equations or branching process theory. Third, many population models linearise to birth–death dynamics near absorbing sets, even when their bulk behaviour is non-linear, making this approach broadly applicable. This reduction enables us to derive theoretical error bounds and practical heuristics for hybrid approximations, providing guidance for threshold selection (e.g., choosing Ω to trade off accuracy and cost). While we focus on birth–death systems, the underlying mathematical framework extends to more general systems beyond those that are inherently birth–death processes.

### Linearisation and the time-inhomogeneous birth–death process

Our analysis begins by partitioning the system’s species into two sets:

- *Scarce species * (*J*): Species i∈J with $X_i \le \Omega$, treated stochastically.
- *Abundant species * (*F*): Species i∈F with $x_i > \Omega$, approximated by deterministic dynamics.

This partitioning mirrors the logic of the JSF hybrid methods. The key insight is that when scarce species populations are small, their dynamics linearise. Assuming Ω is sufficiently small, our analysis relies on the following conditions, which are biologically reasonable near an extinction boundary:

1. *Condition* 1—*Linear reactions dominate:* Reactions involving two or more scarce individuals are rare and their propensities are negligible.
2. *Condition* 2—*One-way coupling:* The influence of scarce species on abundant ones is negligible. The abundant species thus evolve independently according to $\dot{\textbf{x}}_F = f_F(\textbf{x}_F, t)$, creating a time-varying environment for the scarce species.
3. *Condition 3—No external input:* For simplicity, we assume no external immigration for scarce species. The framework can be straightforwardly extended to include this as needed.

These conditions constrain the *error-bound derivation*, not the JSF method itself; JSF admits fully bidirectional coupling in simulation. Conditions 1 and 2 are also asymptotically self-consistent near extinction. The neglected nonlinear propensities and the feedback from $\textbf{X}_J$ to $\textbf{x}_F$ are both $O(\Vert \textbf{X}_J\Vert )$ at the per-capita level, so they vanish in the regime that controls the extinction probability. The bound is most useful when a small subset of species is rare against a much larger, near-deterministic background: rare-mutant invasion, late-stage viral clearance, pathogen extinction in a host at carrying capacity, minority-species extinction in a large ecosystem, the die-out regime of stochastic SIR dynamics. Sustained feedback from scarce to abundant species, or prolonged near-critical phases where r(t)≈0, lie outside this analysis. Section 6 returns to these.

In general, the per-capita birth and death rates for the scarce species depend on the full hybrid state $\textbf{z} = (\textbf{X}_J, \textbf{x}_F)$. For each scarce species i∈J, define

- $\mu _i(\textbf{z})$: per-capita death rate of type *i*;
- $\Lambda _{\ell i}(\textbf{z})$: per-capita rate at which a parent of type *i* produces an offspring of type ℓ.

#### Condition 1

*(linear reactions dominate)* says reactions involving two or more scarce individuals are rare. Under standard mass-action scaling, their propensities are $O(\Vert \textbf{X}_J\Vert ^2)$, so their *per-capita* contributions are $O(\Vert \textbf{X}_J\Vert )$ and vanish as $\Omega \rightarrow 0$.

7 $$\begin{aligned} \mu _i(\textbf{z}) \;=\; \mu _i^{(0)}(\textbf{x}_F) \;+\; O\!\left( \Vert \textbf{X}_J\Vert \right) ,\qquad \Lambda _{\ell i}(\textbf{z}) \;=\; \Lambda _{\ell i}^{(0)}(\textbf{x}_F) \;+\; O\!\left( \Vert \textbf{X}_J\Vert \right) . \end{aligned}$$

#### Condition 2

*(one-way coupling)* fixes $\textbf{x}_F(t)$ as a deterministic trajectory. Neglecting the $O(\Vert \textbf{X}_J\Vert )$ terms as $\Omega \rightarrow 0$ yields time-dependent per-capita rates that depend only on the abundant-species environment:

8 $$\begin{aligned} \mu _i(t)\;\equiv \;\mu _i^{(0)}\!\big (\textbf{x}_F(t)\big ), \qquad \Lambda _{\ell i}(t)\;\equiv \;\Lambda _{\ell i}^{(0)}\!\big (\textbf{x}_F(t)\big ). \end{aligned}$$

With these rates, each scarce individual evolves independently (dies at rate $\mu _i(t)$; produces an ℓ-type offspring at rate $\Lambda _{\ell i}(t)$), so $\textbf{X}_J(t)$ is a time-inhomogeneous multi-type branching (birth–death) process (Kendall 1948; Dewitt et al. 2024).

### Calculating extinction probabilities

The linearised birth–death structure allows us to compute extinction probabilities using branching process theory. We begin with the general multi-type framework and then specialise to the single-species case, which will be the focus of our subsequent analysis.

Let $\textbf{q}(s) = (q_i(s))_{i \in J}$ be the vector where $q_i(s)$ is the probability that the lineage (all descendants) of a single individual of species i∈J present at time *s* is extinct by the final time *T*. This vector satisfies a backward Kolmogorov system (see Online Appendix S.I.1):

9 $$\begin{aligned} \frac{d}{ds}\,\textbf{q}(s) = \boldsymbol{\mu }(s) \circ \big (\textbf{q}(s) - \textbf{1}\big ) + \textbf{q}(s)\circ \!\Big [\Lambda (s)^{\!\top }\big (\textbf{1}-\textbf{q}(s)\big )\Big ], \qquad \textbf{q}(T)=\textbf{0}, \end{aligned}$$

where $\boldsymbol{\mu }(s)$ collects the death rates, ∘ denotes the Hadamard (element-wise) product, and the (ℓ,i) entry of Λ is the per-capita rate at which a parent of type *i* produces an offspring of type ℓ.

The total probability of extinction for a population with integer initial counts $\textbf{n}_s=(n_{s,i})_{i\in J}$ at time *s* and horizon *T* is then

10 $$\begin{aligned} P_{\text {ext}}(\textbf{n}_s, s \mid T) = \prod _{i \in J} q_i(s)^{\,n_{s,i}} . \end{aligned}$$

In particular, for initial counts $\textbf{n}_0=(n_{0,i})_{i\in J}$ at time s=0,

11 $$\begin{aligned} P_{\text {ext}}(\textbf{n}_0, 0 \mid T) = \prod _{i \in J} q_i(0)^{\,n_{0,i}} . \end{aligned}$$

While this multi-type formulation is general, for many applications the analysis can be simplified by focusing on a single scarce species, *Y* (so $J = \{Y\}$). In this scenario, the mathematical framework reduces from matrices and vectors to simple scalars:

- The birth matrix Λ(t) becomes a 1×1 matrix, represented by the scalar per-capita birth rate λ(t).
- The death rate vector $\boldsymbol{\mu }(t)$ becomes a single scalar per-capita death rate μ(t).

As before, these rates, $\lambda (t) \equiv \lambda (\textbf{x}_F(t))$ and $\mu (t) \equiv \mu (\textbf{x}_F(t))$, depend on the abundant species’ trajectory.

For this single-species case, the extinction probability for a single lineage, denoted $q_Y(s)$, satisfies the scalar backward Riccati equation (forward time *s*):

12 $$\begin{aligned} \frac{dq_Y}{ds} = \mu (s)\big (q_Y - 1\big ) + \lambda (s)\big (q_Y - q_Y^2\big ), \qquad q_Y(T) = 0. \end{aligned}$$

Given an initial population $Y(0) = n_0 \in \mathbb {N}$, the total probability of extinction by time *T* is found by compounding these independent lineage probabilities:

13 $$\begin{aligned} P_{\text {ext}}(n_0, 0 \mid T) = \big [q_Y(0)\big ]^{n_0}. \end{aligned}$$

In Sect. 4, we will use this scalar Riccati equation to derive error bounds; the multi-type framework proceeds analogously via Eq. (9).

## Error analysis for hybrid method

### Framing the analysis: the collapse scenario

To analyse the error of the JSF hybrid method with a given Ω (recalling that we adopt a common threshold Ω across species, i.e., $\Omega _i = \Omega$ for all *i*), we first consider the initial state of the focal species *Y*. There are two primary biological scenarios:

- *The introduction scenario*, where a species starts at a low count (Y(0)<Ω), such as during the initial phase of an infection or the arrival of an invasive species.
- *The collapse scenario*, where a previously abundant population declines to the threshold, $Y(s_0) = \Omega$. This represents events such as population crashes or disease clearance.

Our analysis will focus on the collapse scenario, which captures the essential error-generating mechanism. To see why, consider that in the introduction scenario, the exact stochastic model and the JSF hybrid model are identical as long as the population remains below the threshold, $Y(t) \le \Omega$. No error can accumulate during this period. If the population hits the threshold at some time $\tau _\Omega < T$, the strong Markov property (Øksendal 2003) allows us to restart the analysis from that point. The problem then becomes equivalent to a collapse scenario starting at time $\tau _\Omega$, and error bounds derived for the collapse scenario apply directly, with rates evaluated at the crossing time $\tau _\Omega$.

Focusing on the collapse scenario is powerful because it frames the problem in terms of the immediate dynamics at the boundary. With the population starting at Y(0)=Ω, its subsequent evolution is dictated by the net growth rate, $r(t) = \lambda (t) - \mu (t)$. A negative rate (r(t)<0) creates a “drift toward extinction,” tending to keep the population in the stochastic regime, where the models agree. Conversely, a positive rate (r(t)>0) creates a “drift away from extinction”, pushing the population into the deterministic regime, where approximation errors are generated.

This birth–death perspective, introduced in Sect. 3 and here manifest in the interplay between birth rate λ(t), death rate μ(t), and the threshold Ω, is crucial for understanding how and when the JSF approximation introduces significant error, as we develop in the following subsections.

### Error metric for hybrid approximations

To quantify the accuracy of the JSF approximation for a given threshold Ω, we define the approximation error as the absolute difference between the total extinction probabilities computed by the exact and hybrid models. We denote the exact probability as $P_{\text {ext}}(\textbf{x}_0, 0 | T)$ and the JSF approximation as $P_{\text {ext}}^\textrm{JSF}(\textbf{x}_0, 0 | T)$.

Starting from an initial condition $\textbf{x}_0$ at time s=0, the error over a time horizon *T* is:

14 $$\begin{aligned} \Delta _\Omega (\textbf{x}_0, T) := \big | P_{\text {ext}}(\textbf{x}_0, 0 | T) - P_{\text {ext}}^\textrm{JSF}(\textbf{x}_0, 0 | T) \big |. \end{aligned}$$

This error metric captures how the choice of threshold Ω affects the accuracy of extinction probability estimates, providing the foundation for principled threshold selection. When the initial condition is clear from context (e.g., Y(0)=Ω in the collapse scenario), we write simply $\Delta _\Omega (T)$.

### Critical time regimes and error decomposition

The key insight for our error analysis is that the discrepancy between the exact stochastic model and the JSF hybrid method, $\Delta _\Omega (T)$, arises only when the focal species *Y* up-crosses the threshold Ω. The first up-crossing time, $\tau _\uparrow$, occurs where $\tau _\uparrow := \inf \{t \ge 0: Y(t) = \Omega + 1\}$. At this moment, the hybrid model switches to a deterministic ODE, while the true process remains stochastic. The consequences of this divergence depend critically on the system’s dynamics at the time of the up-crossing.

The behaviour of the birth–death process is governed by the net growth rate $r(t) = \lambda (t) - \mu (t)$. This allows us to define two crucial time points:

- *The critical time,* $t_c$, is the point where the dynamics shift from being death-dominated (r(t)<0) to birth-dominated (r(t)≥0). It is defined as:
  15 $$\begin{aligned} t_c := \inf \{t \ge 0 : r(t) \ge 0\}. \end{aligned}$$
  Excursions above Ω before $t_c$ tend to be corrected by the negative drift, whereas those occurring after may persist.
- *The point of no return,* $s_*$, represents the latest moment an up-crossing can occur and still be expected to return below Ω deterministically before the drift turns favourable at $t_c$. It is defined by the solution to (see Online Appendix S.I.3):
  16 $$\begin{aligned} \int _{s_*}^{t_c} r(u) \, du = \log \left( \frac{\Omega }{\Omega + 1}\right) . \end{aligned}$$

**Fig. 1:**
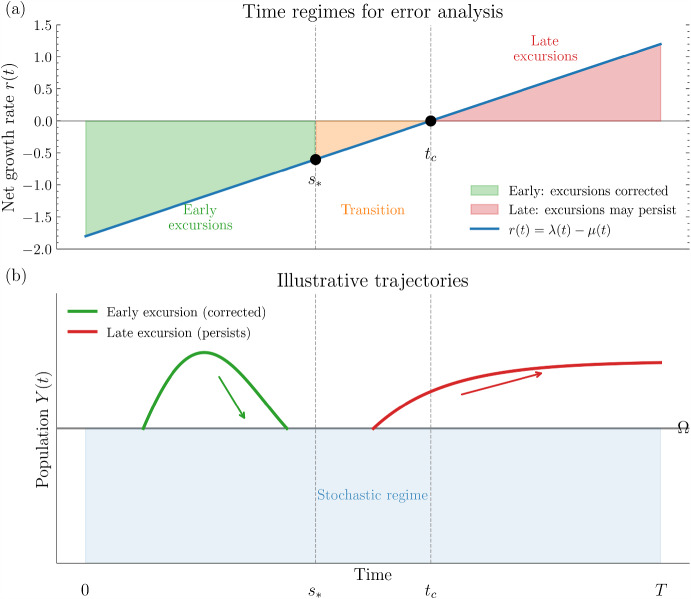
Illustration of the critical time $t_c$ and point of no return $s_*$. **a** The net growth rate $r(t) = \lambda (t) - \mu (t)$ transitions from negative (death-dominated) to positive (birth-dominated) at $t_c$. The point of no return $s_*$ marks the latest time an up-crossing can occur and still be expected to return below Ω before $t_c$. **b** Illustrative trajectories: early excursions (green) are corrected by negative drift, while late excursions (red) persist due to favourable growth conditions

These time points, illustrated in Fig. 1, allow us to partition the total error based on $\tau _\uparrow$. The formal steps for this decomposition are provided in Online Appendix S.I.3. This end result separates the error into two conceptually distinct early/late components:

17 $$\begin{aligned} \Delta _\Omega (T) \le \underbrace{\mathbb {E}\left[ [q_Y(\tau _\uparrow )]^{\Omega +1} \textbf{1}\{\tau _\uparrow< s_{*}\} \right] }_{\text {Early Excursion Error}} + \underbrace{\mathbb {E}\left[ [q_Y(\tau _\uparrow )]^{\Omega +1} \textbf{1}\{s_{*} \le \tau _\uparrow < T\} \right] }_{\text {Late Excursion Error}}. \end{aligned}$$

The subsequent analysis will focus on bounding each of these terms.

### A rigorous but impractical bound

A full theoretical bound on the error can be derived by combining the contributions from both early and late excursions. The detailed derivation is presented in Online Appendix S.I.3. In brief, the approach decomposes each stochastic excursion into a deterministic drift component and a martingale fluctuation, bounds the duration of excursions using a drift argument, and then applies the Burkholder–Davis–Gundy (BDG) inequality to control the martingale deviations via their predictable quadratic variation. This yields the following result, cf. Eq. (17):

18 $$\begin{aligned} \Delta _\Omega (T) \le \underbrace{\left( \int _0^{s_*}\Omega \lambda (t)\,dt\right) \frac{L_{\text {eff}}}{\rho } \frac{\Omega +1}{\Omega } C_{\text {BDG}} \sqrt{V_\star }}_{\text {Early Excursion Error}} + \underbrace{\int _{s_*}^T w(s) [q_Y(s)]^{\Omega +1}\,ds}_{\text {Late Excursion Error}}. \end{aligned}$$

where: $\rho := \inf _{t\in [0,s_*]} \{-r(t)\}$ is the uniform lower bound on the negative drift; $L_{\textrm{eff}}:= \sup _{s\in [0,s_*]} |p'(s)|$ is the Lipschitz constant of the extinction probability $p(t):= q_Y(t)^{\Omega }$; $C_{\textrm{BDG}}$ is the Burkholder–Davis–Gundy constant; $V_\star := \sup _{s\in [0,s_*]}\int _s^{\sigma (s)} [\lambda (u)+\mu (u)]\,y^{(s)}(u)\,du$ bounds the predictable quadratic variation over early excursions, with $y^{(s)}(u)$ the deterministic trajectory and σ(s) its return time to Ω; and $w(s):= \Omega \lambda (s) \exp \bigl (-\Omega \int _0^s \lambda (u) \, du\bigr )$ is the up-crossing time density. See Online Appendix S.I.3 for full derivations.

While mathematically rigorous, this bound has significant practical limitations that stem from the early excursion term. First, its implementation is computationally demanding, requiring careful numerical solutions, the evaluation of suprema and infima over time intervals ($L_{\text {eff}}, \rho$), and the estimation of martingale quadratic variations ($V_\star$). Second, and more critically, the bound is often overly conservative, yielding impractically large estimates (≫1). This is a direct result of compounding several worst-case estimates: the maximum rate of change of the extinction probability ($L_{\text {eff}}$), the weakest possible negative drift (ρ), the largest possible stochastic fluctuation ($V_\star$), and the use of a universal $C_{\textrm{BDG}}$ which controls the worst-case supremum of a martingale from its quadratic variation rather than the typical behaviour. To take a specific example, computing $L_{\textrm{eff}}$ using a global supremum over the entire early window $[0, s_*]$ overweights periods when the process is far from Ω, even though sensitivity is primarily needed during the rare moments when trajectories remain near the threshold. The use of global bounds on quantities that are only relevant during brief threshold-crossing events leads to a significant overestimation of the error.

Given these limitations, the primary value of the full bound is theoretical. For practical applications, a more direct and computationally tractable approach is needed. This motivates the development of a heuristic that isolates the dominant source of error, which we present in the next section.

### A practical heuristic for error estimation

The rigorous bound derived in the previous section, while theoretically complete, is often computationally intensive and overly conservative for practical use. This motivates a simpler approach that isolates the dominant source of error.

The key insight is that in many systems, the error budget is dominated by the late excursion term. When the death rate significantly exceeds the birth rate in the early phase (r(t)≪0 for $t < s_*$), any stochastic excursions above the threshold Ω are corrected rapidly by the strong negative drift. Consequently, the contribution from the early excursion error is often negligible.

This allows us to approximate the total error by retaining only the late term. Bounding the integral for the late excursion probability leads to a simple and effective heuristic (see Online Appendix S.I.3.4 for derivation):

19 $$\begin{aligned} \Delta _\Omega (T) \lesssim [q_Y(s_*)]^{\Omega +1}. \end{aligned}$$

We can interpret this formula in a straightforward manner: the dominant error is controlled by the probability of extinction as seen from the “point of no return”, $s_*$ (see Fig. 1). This is the latest moment at which a trajectory that has crossed into the deterministic regime can still be expected to return below Ω before the system’s dynamics become favourable for persistent growth.

Algorithm 1 outlines the straightforward procedure for calculating this heuristic bound.

**Algorithm 1:**
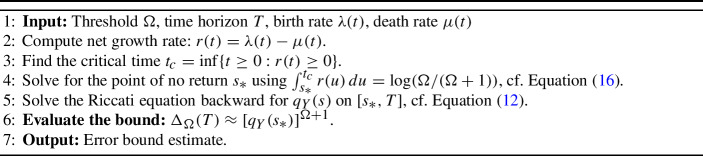
Calculating the Heuristic error bound

## Case study: the stochastic Lotka–Volterra model

We now demonstrate the practical application of our birth–death framework by analysing a classic predator–prey system with stochastic Lotka-Volterra (LV) dynamics. This system is a suitable case study because it is simple enough to admit explicit analytical solutions while being rich enough to exhibit all the key phenomena of our general theory: time-varying birth–death rates, critical time transitions, and the interplay between stochastic and deterministic dynamics. Moreover, the LV model is sufficiently well-known that readers can focus on the methodology rather than the biological details.

### The stochastic Lotka–Volterra model, scarce prey

#### Model definition

Consider a two-species system with prey $X_1$ (scarce, $J = \{1\}$) and predators $X_2$ (abundant, $F = \{2\}$). The reaction network with rates $\alpha , \beta , \gamma > 0$ is:

20 $$\begin{aligned} \text {(Prey birth)} &  X_1&\xrightarrow {\alpha } 2X_1, \end{aligned}$$

21 $$\begin{aligned} \text {(Predation)} &  X_1 + X_2&\xrightarrow {\beta } 2X_2, \end{aligned}$$

22 $$\begin{aligned} \text {(Predator death)} &  X_2&\xrightarrow {\gamma } \emptyset . \end{aligned}$$

When prey is scarce, predation events contribute little to the predator population compared to predator death, which dominates predator dynamics. Under our linearisation assumption (Sect. 3), predator dynamics decouple from prey when prey counts are small, yielding the independent evolution:

23 $$\begin{aligned} \dot{x}_2(t) = -\gamma x_2(t), \qquad \Rightarrow x_2(t) = x_{2,0} e^{-\gamma t}. \end{aligned}$$

This exponential decay of predators directly determines the prey birth–death rates. The prey birth reaction contributes constant rate α to λ(t), while predation contributes time-varying rate $\beta x_2(t)$ to μ(t). Thus (cf. Section 3.2):

24 $$\begin{aligned} \lambda (t) = \alpha , \qquad \mu (t) = \beta x_{2,0} e^{-\gamma t}, \qquad r(t) = \alpha - \beta x_{2,0} e^{-\gamma t}. \end{aligned}$$

**Fig. 2:**
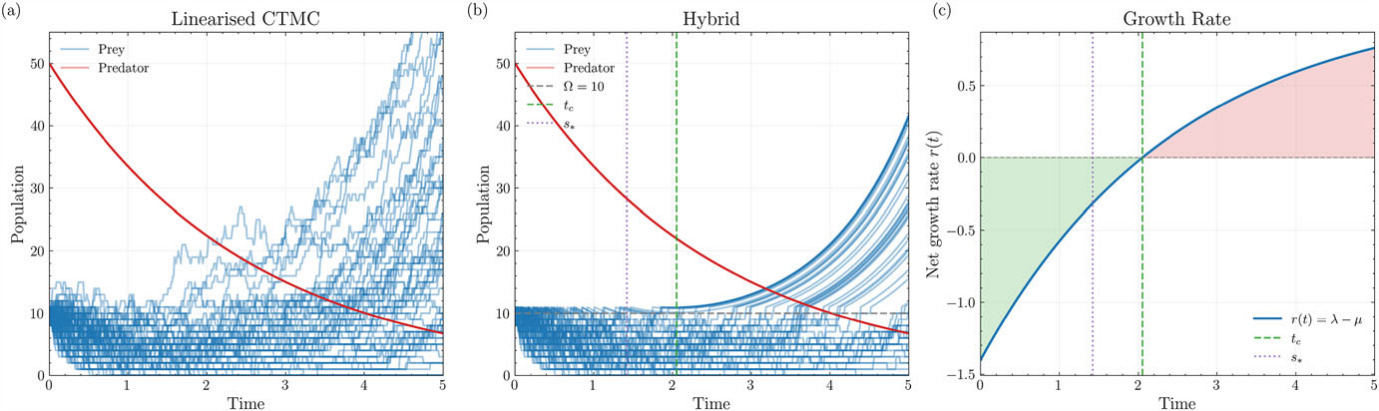
Comparison of the linearised CTMC reference and the JSF hybrid for the Lotka–Volterra predator–prey model. Parameters: α=1.10, β=0.05, γ=0.4, initial conditions $(x_{1,0}, x_{2,0}) = (10, 50)$. Panel **a** shows 1, 000 trajectories of the linearised CTMC reference: prey (blue) evolves stochastically via the SSA and predator (red) follows the deterministic ODE $\dot{x}_2 = -\gamma x_2$, consistent with the linearisation of Sect. 3. Panel **b** shows 1, 000 trajectories of the full JSF hybrid with switching threshold Ω=10: prey is simulated stochastically while $X_1\le \Omega$ and deterministically otherwise, predator as in panel (**a**). Panel **c** shows the net growth rate $r(t)=\lambda (t)-\mu (t)$, with the critical time $t_c$ (dashed, $r(t_c)=0$) and point of no return $s_*$ (dotted) annotated; the green region is death-dominated (r<0) and the red region is birth-dominated (r>0)

**Fig. 3:**
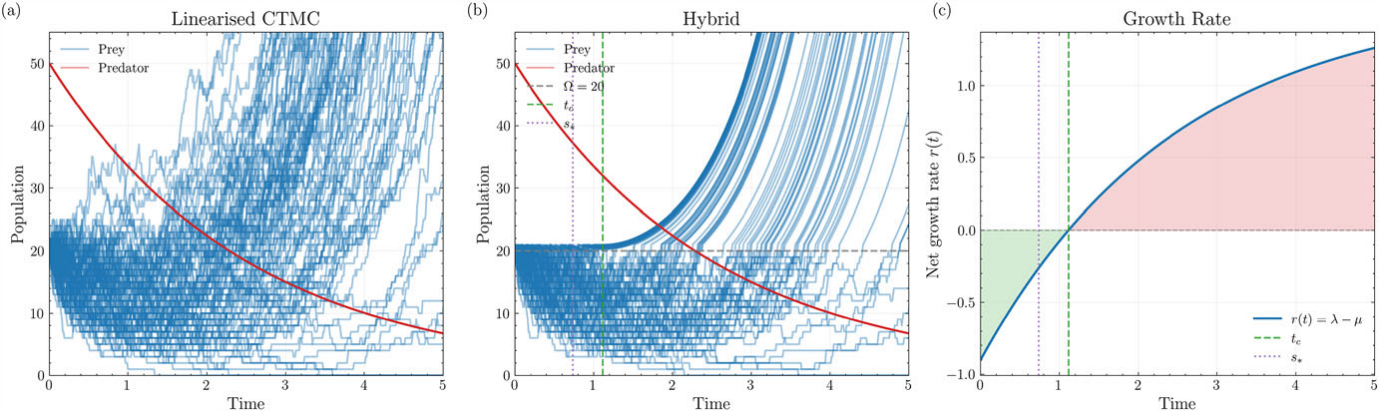
As Fig. 2, with increased prey birth rate α=1.60. The higher birth rate lowers extinction probability and shifts both $t_c$ and $s_*$ earlier

Figures 2 and 3 show two example simulations of the LV model. We simulate the system under two configurations of the JSF method: a *linearised CTMC reference*, in which the scarce prey is evolved stochastically while the abundant predator follows its deterministic ODE (Eq. (23))—consistent with the linearisation assumptions of Sect. 3 against which our error bound is derived—and the full *JSF hybrid* with switching threshold Ω=10, in which the prey switches between stochastic and deterministic regimes at Ω. Each configuration aggregates 1, 000 realisations of the stochastic process.

#### Numerical validation

All quantities from our general framework – extinction probabilities $q_Y(\cdot )$, critical times $t_c$ and $s_*$, and error bounds – follow by direct substitution of the rates Eq. (24) and applying Algorithm 1. Explicit expressions for the relevant quantities are given in Online Appendix S.I.5.

To validate our theoretical framework, we conduct Monte Carlo simulations comparing exact stochastic trajectories with JSF hybrid approximations across a range of threshold parameters. This computational experiment serves two purposes: it demonstrates the practical utility of our error bounds and provides empirical validation of the birth–death theoretical predictions.

We consider the Lotka-Volterra system with parameters α=1.10, β=0.04, γ=0.1, initial conditions $(x_{1,0}, x_{2,0}) = (10, 50)$, and time horizon T=7. For each threshold value $\Omega \in \{5, 10, 15, 20, 25, 30, 35\}$, we perform 32, 000 independent simulations using both the linearised CTMC reference (scarce prey simulated by the SSA with the abundant predator evolving deterministically, cf. Sect. 3) and the JSF hybrid method. From these simulations, we compute:

- *Empirical error:* $\hat{\Delta }_\Omega (T) = |\hat{P}_{\text {ref}} - \hat{P}_{\text {JSF}}|$, where $\hat{P}$ denotes the empirical extinction probability as determined by Monte Carlo simulation (subscript “ref” denotes the linearised CTMC reference)
- *Confidence intervals:* Wilson score intervals accounting for binomial sampling variability
- *Practical heuristic bound:* $[q_Y(s_*)]^{\Omega +1}$ from our late-only approximation (Eq. 19), computed by solving the Riccati equation and finding the critical time $s_*$ (see Online Appendix S.I.5)

**Fig. 4:**
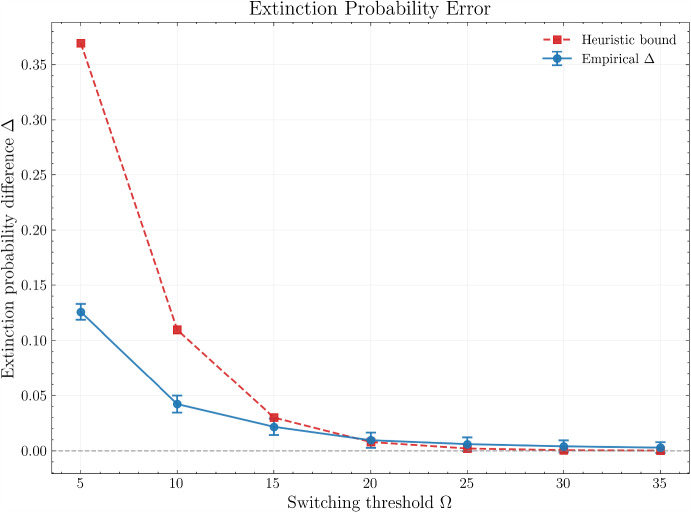
Validation of the practical heuristic bound for the Lotka-Volterra system. Blue circles show empirical error $\Delta _\Omega (T)$ between the linearised CTMC reference (scarce prey via SSA, abundant predator via ODE, cf. Sect. 3) and the JSF hybrid simulations (32, 000 replications each), with Wilson score confidence intervals. Red dashed line shows the late-only heuristic bound $[q_Y(s_*)]^{\Omega +1}$. The simplified bound provides a reliable upper envelope, demonstrating the effectiveness of the practical approximation. Parameters: α=1.10, β=0.04, γ=0.1, $(x_{1,0}, x_{2,0}) = (\Omega , 40)$, T=7

Figure 4 presents the key results. The empirical error (blue circles with confidence intervals) exhibits the expected monotonic decrease with increasing Ω. As discussed in our theoretical development, when Ω is small, the hybrid model operates primarily in the deterministic regime, leading to larger discrepancies with the exact stochastic behaviour. As Ω increases, the models converge since both treat the focal species stochastically throughout most trajectories.

Critically, our practical heuristic bound (red dashed line) provides a reliable upper envelope for the empirical error across all threshold values. This simplified bound, which neglects the computationally intensive early-term contributions and focuses on the dominant late-excursion errors, successfully captures both the magnitude and the functional dependence on Ω. The validation demonstrates that the late-only approximation is indeed sufficient for practical threshold selection in this birth–death system.

#### Parameter space exploration

The previous analysis focused on a particular instance of the Lotka-Volterra system with fixed parameters. To assess the robustness of our theoretical framework and the practical heuristic bound across different dynamical regimes, we now systematically explore the parameter space.

We vary the prey birth rate $\alpha \in [1.0, 1.2]$ and predation rate $\beta \in [0.04, 0.06]$ while holding the predator death rate fixed at γ=0.4, with initial conditions $(x_{1,0}, x_{2,0}) = (\Omega , 40)$ and time horizon T=7. This parameter range spans systems from near-certain extinction (low α, high β) to high survival probability (high α, low β). For each of the 36 parameter combinations on a 6×6 grid, we compute the heuristic monotonicity bound for Ω=10 and perform 32, 000 independent simulations each of the linearised CTMC reference and JSF hybrid methods. We calculate the empirical extinction probability gap $\Delta _{\text {emp}} = p_{\text {stochastic}} - p_{\text {hybrid}}$ and compare it to the deterministic theoretical bound, computing the margin (bound − empirical) and its statistical significance via z-score (margin / standard error).

**Fig. 5:**
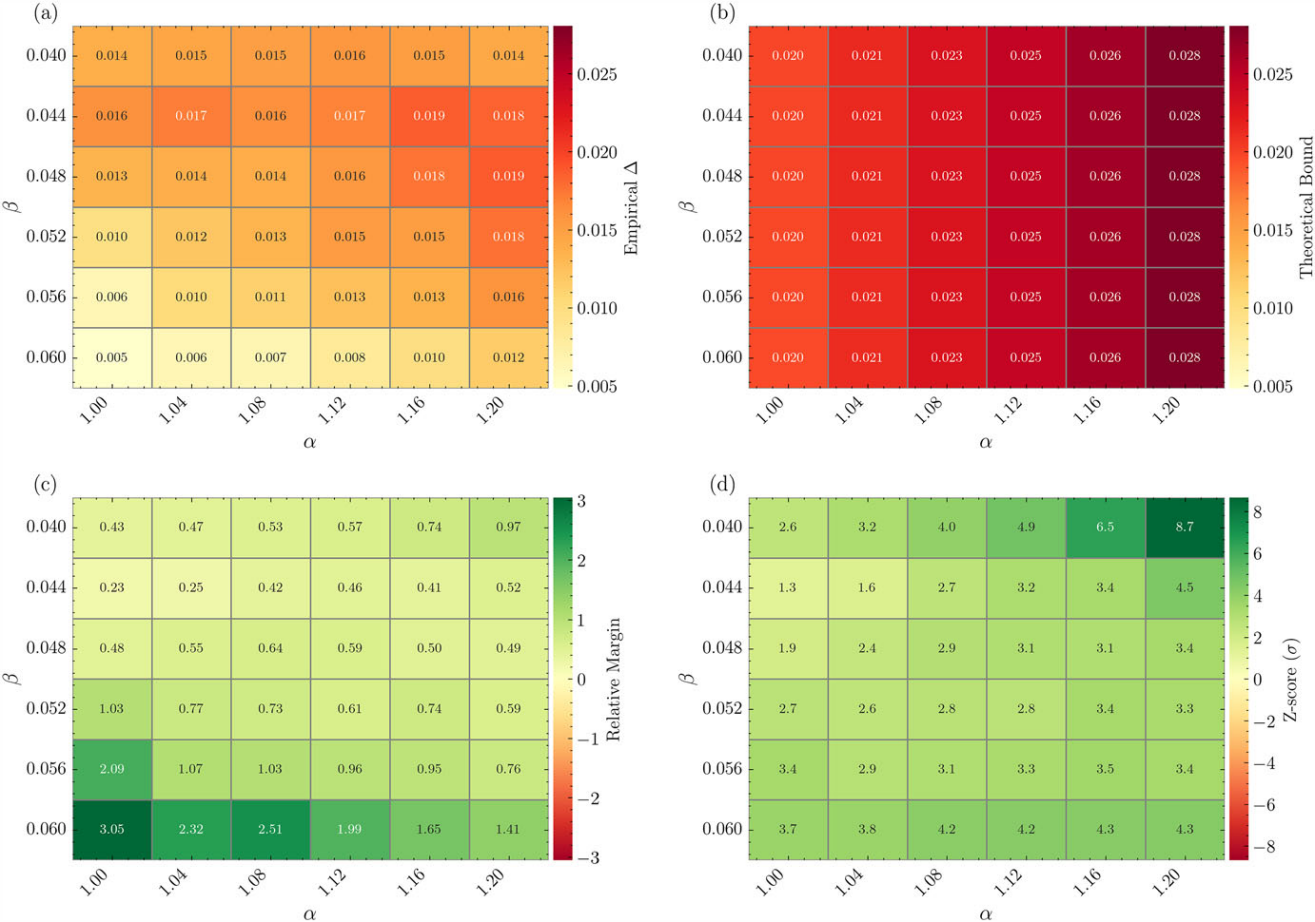
Parameter space exploration for Ω=10 across prey birth rate α and predation rate β. **a** Empirical extinction probability gap $\Delta _{\text {emp}}$ from 32, 000 simulations per grid point. **b** Deterministic theoretical bound values showing monotonic dependence on α but near-invariance to β. **c** Margin (bound slack) with green indicating positive margins; the black dashed contour at margin=0 indicates the bound-violation boundary. **d** Statistical significance (z-score = margin/error) with green indicating the bound holds and red indicating violations. All 36 parameter combinations show positive margins, with z-scores ranging from 1.3σ to 8.7σ, validating the theoretical framework across diverse dynamical regimes from high-extinction to high-survival scenarios

Figure 5 presents the results across four complementary views of the parameter space. The practical heuristic bound holds reliably across the parameter range tested, with margins ranging from 0.004 to 0.016 and z-scores from 1.3σ to 8.7σ. All 36 grid points exhibit strictly positive margins, and every z-score is at least 1.3σ above zero, providing strong empirical support for the bound across the tested parameter range. While the theoretical bound is deterministic, the empirical gap has sampling uncertainty from finite simulations (Bernoulli trials), making the z-score essential for assessing whether observed margins reflect true bound slack versus measurement noise.

Notably, the theoretical bound exhibits near-perfect linear growth with increasing prey birth rate α (correlation of 0.9998, from ≈0.020 at α=1.0 to ≈0.028 at α=1.2), reflecting increased coupling strength between the exact and hybrid dynamics in more favourable demographic conditions. This dependence on α can be understood practically: larger prey birth rates increase the net growth rate $r = \alpha - \beta x_2$ of the prey population, amplifying the rate at which stochastic and deterministic trajectories can diverge before reaching the extinction boundary. In contrast, the bound remains nearly invariant to changes in predation rate β, varying by less than 0.001 within each α slice. The empirical gap shows similar trends but with greater sensitivity to β, resulting in margin variation across both dimensions. The tightest margins occur at low α=1.0 where the bound approaches the empirical gap most closely, while the loosest margins (≈0.016) appear at intermediate parameter values where the empirical gap is smallest relative to the bound.

## Discussion and conclusions

We have demonstrated that principled threshold selection for JSF-type hybrid methods can be achieved through birth–death analysis near extinction boundaries. Hybrid multiscale schemes, like JSF, promise CTMC fidelity where discreteness matters and ODE speed where it does not, but the practical difficulty in deployment (i.e. choosing the switching threshold(s) Ω) has remained largely arbitrary. Our framework reframes this challenge as an extinction-accuracy budgeting problem with explicit, computable bounds.

By linearizing near absorbing sets and exploiting birth–death structure, we separated the approximation error into two components: (i) *early excursions* under negative drift (r(t)<0), which are typically self-correcting and contribute minimally to the error budget, and (ii) *late excursions* once the net growth becomes nonnegative (r(t)≥0), which dominate bias in extinction observables. This decomposition yields both a rigorous theoretical bound and, crucially, a compact heuristic that requires only solving a single Riccati equation rather than thousands of Monte Carlo simulations.

Our validation with the case study involving the Lotka-Volterra system confirms that the late-only estimate, Eq. (19) reliably captures the main failure mode of JSF for extinction problems. This bound identifies paths that cross into the deterministic regime near or after the critical time $t_c$ when *r*(*t*) changes sign, and thus do not return quickly to the stochastic boundary where discreteness matters. When there is a distinct period of death dominance on $[0,s_*]$ followed by a transition to birth dominance, early excursions are rapidly corrected by negative drift, and the late term controls the bias. In order to choose a value of Ω a user must fix a time horizon *T* and an extinction-probability error tolerance ε, and choose

25 $$\begin{aligned} \Omega \ge \big \lceil \frac{\log \varepsilon }{\log q_Y(s_*)} \big \rceil - 1, \end{aligned}$$

The heuristic approach scales as *O*(*T*) for solving the Riccati equation (Laine and Tomlin 2018) plus $O(\log 1/\delta )$ for the one-dimensional root finding required to determine $s_\star$, where δ is the numerical tolerance used in the bisection method (Burden et al. 2016), see Online Appendix S.I.4. This contrasts with $O(N \times T \times R)$ for Monte Carlo validation with *N* replications, *T* time steps, and *R* reactions per step.

For multiple scarce species i∈J, a union bound over species-specific errors gives

26 $$\begin{aligned} \Delta _\Omega (T) \lesssim \sum _{i \in J} [q_i(s_{*,i})]^{\Omega _i+1}. \end{aligned}$$

This bound holds because the total error cannot exceed the sum of individual species errors. As before, to specify switching thresholds $\Omega _i$, first allocate per-species error budgets $\varepsilon _i$ with $\sum _i \varepsilon _i \le \varepsilon$ and then choose the thresholds $\Omega _i$ accordingly. In practice, it is recommended to start with equal budgets and then tighten those for species with large $q_i(s_{*,i})$ values.

### Limitations and scope of applicability

The heuristic, Eq. (19), relies on three modelling assumptions that underpin the linearisation (Sect. 3.1): scarce–scarce interactions are $O(\Vert \textbf{X}_J\Vert ^2)$ and so negligible near extinction; the coupling from abundant to scarce species dominates the reverse coupling in the low-count regime; and scarce species receive no external immigration (or immigration is modelled explicitly). These bind the error-bound derivation, not the JSF simulation: JSF is indifferent to the coupling structure, and the assumptions are simply the minimal conditions under which the scarce-species dynamics near extinction reduce to a time-inhomogeneous birth–death process with a tractable Riccati equation. The first and second are asymptotically self-consistent. Both neglected contributions are $O(\Vert \textbf{X}_J\Vert )$ at the per-capita level and vanish in the regime that determines extinction. The framework then applies to systems in which a rare subset lives inside a large, near-deterministic background, for example rare-mutant invasion, late-stage viral clearance, pathogen extinction in a host at carrying capacity, and SIR die-out.

Even within scope, the heuristic’s tightness depends on whether the dynamics separate cleanly into a death-dominated early phase and a birth-dominated late phase. The Lotka–Volterra case study (Sect. 5.1) sits in this regime: the one-way approximation enters when we drop the predation contribution to predator dynamics in Eq. (23), leaving predators to decay exponentially. The dropped term is $O(X_1)$, so this is justified when prey are scarce, but it would need to be retained in systems where scarce prey sustain an otherwise weakly-coupled predator population, or more generally where there is strong feedback from $\textbf{X}_J$ onto $\textbf{x}_F$.

The heuristic can also under-estimate the error in two further regimes. If the system has a sustained near-critical phase where r(t)≈0, early excursions are no longer rapidly corrected and the early term in the rigorous bound (Eq. (18)) cannot be neglected. If scarce–scarce interactions are strong (e.g., cooperative effects, Allee dynamics), neglecting the $O(\Vert \textbf{X}_J\Vert ^2)$ propensities changes the extinction dynamics qualitatively rather than only quantitatively. In both cases, one would need to use the full bound in Eq. (18) or apply a conservative margin to the heuristic.

### Broader implications and future directions

The error-control framework is method-agnostic. The heuristic bound $[q_Y(s_*)]^{\Omega +1}$ depends only on the birth and death rates $\lambda (t),\mu (t)$ of the scarce species near the extinction boundary, the time horizon *T*, and the threshold Ω. It does not depend on the rule by which a hybrid simulator decides when to switch between stochastic and deterministic regimes, nor on how the continuous component is integrated between jumps. The same inequality therefore applies, essentially unchanged, to the multi-level schemes of Winkelmann and Schütte (2017, 2020), the regime-conversion method of Kynaston et al. (2023), partitioned leaping (Cao et al. 2005), and the PDMP-style hybrids of Crudu et al. (2009); Marchetti et al. (2016); Alfonsi et al. (2004), provided the simulator preserves the stochastic birth–death dynamics of the scarce species below the threshold, as all threshold-based hybrids do by construction. JSF is the simulator we use here, but the current analysis is not tied to it: the contribution of this work is a recipe for equipping any threshold-based hybrid simulation method with an *a priori* error bound on extinction observables. Beyond the choice of simulator, the birth–death reduction is a template for accuracy control in any rare-event problem where linearisation near a critical point is valid. Three extensions follow naturally. Time- or state-dependent thresholds Ω(t) that grow as $t \rightarrow t_c$ would suppress late-excursion bias. Modified backward equations would carry the analysis to first-passage times, outbreak probabilities, and other terminal functionals. The same construction should also adapt to non-extinction observables such as moments and steady-state distributions .

In practice the model parameters $\boldsymbol{\theta }$ are often unknown and inferred from data, while our threshold rule uses specific parameter values to compute $s_*(\boldsymbol{\theta })$ and $q_Y(s_*;\boldsymbol{\theta })$. This is appropriate, since the correct treatment of the system depends on $\boldsymbol{\theta }$, but it couples inference and hybridisation: the choice of Ω affects computational cost, and a too-small Ω can bias the likelihood or summary statistics used for calibration. We leave this for future work.

## Supplementary Information

Below is the link to the electronic supplementary material.

Supplementary file 1 (pdf 333 KB)

## Data Availability

Data sharing is not applicable to this article as no datasets were generated or analysed during the current study.

## Footnotes

1 https://dgermano8.github.io/JSF/
