# Supplementary material for: Multiscale modelling of birth–death processes
Source: J Math Biol. 2026 Aug 3;93(2):30. doi: 10.1007/s00285-026-02448-5 (PMC13433386; doi:10.1007/s00285-026-02448-5)
Supplement: Supplementary file 1 [file 285_2026_2448_MOESM1_ESM.pdf]

S.I.1 Backward equation for extinction probabilities

In this section, we derive the backwards Kolmogorov system given in Equation (9). Consider a time-inhomogeneous multitype Markov branching process on types J. At time s, a single type-i individual: (i) dies at rate $\mu_i(s)$; (ii) gives birth to one additional individual of type j at rate $\Lambda_{ij}(s)$. Let $\mathbf{q}(s) = (q_i(s))_{i\in J}$ be the vector whose ith component is the probability that a lineage started by a single type-i individual at time s is extinct by final time T, with terminal condition $q_i(T) = \mathbb{1}\{i \notin J\} = 0$.

By the backward Kolmogorov equation, conditioning on the first event in $(s, s + \mathrm{d}s)$,

$$q_i(s) = \mu_i(s)\,\mathrm{d}s \cdot 1 \;+\; \sum_{j\in J} \Lambda_{ij}(s)\,\mathrm{d}s\; q_i(s+\mathrm{d}s)\, q_j(s+\mathrm{d}s) \;+\; \Big(1 - \mu_i(s)\mathrm{d}s - \sum_j \Lambda_{ij}(s)\mathrm{d}s\Big)\, q_i(s+\mathrm{d}s) + o(\mathrm{d}s).$$

Subtracting $q_i(s + \mathrm{d}s)$, dividing by $\mathrm{d}s$ and letting $\mathrm{d}s \downarrow 0$ gives

$$\frac{d}{ds} q_i(s) \;=\; \mu_i(s)\big(q_i(s) - 1\big) \;+\; q_i(s)\sum_j \Lambda_{ij}(s) \;-\; q_i(s)\sum_j \Lambda_{ij}(s) q_j(s).$$

In vector form, using the Hadamard (element-wise) product $\circ$ and $\mathbf{1}$ for the all-ones vector,

$$\frac{d}{ds}\mathbf{q}(s) = \boldsymbol{\mu}(s) \circ \big(\mathbf{q}(s) - \mathbf{1}\big) + \big(\Lambda(s)\mathbf{1}\big) \circ \mathbf{q}(s) - \mathbf{q}(s) \circ \big(\Lambda(s)\mathbf{q}(s)\big),$$

with terminal condition $\mathbf{q}(T) = \mathbf{0}$. For a single type with rates $\lambda(s)$ and $\mu(s)$ this reduces to the Riccati ODE $\dot{q} = \mu(s)\,(q - 1) + \lambda(s)\, q\,(1 - q)$.

S.I.2 The Jump–Switch–Flow algorithm

This appendix provides a self-contained mathematical and algorithmic description of the Jump–Switch–Flow (JSF) method used throughout the main text. The presentation adapts the formulation of Germano et al. [2024]; readers seeking further details, implementation notes, or open-source code are directed there.

S.I.2.1 State space and partition

Consider a reaction network with n compartments $\mathbf{V}(t) = (V_1(t), \ldots, V_n(t))$ and m reactions $\{\mathcal{R}_k\}_{k=1}^{m}$, each with propensity $\lambda_k(\mathbf{V})$ and stoichiometric vector $\boldsymbol{\eta}_{\cdot k} \in \mathbb{Z}^n$. For each compartment i, JSF partitions its domain at a user-chosen threshold $\Omega_i \in \mathbb{Z}_{\geq 0}$ into a discrete part and a continuous part:

$$\mathcal{V}_{\Omega_i} \;=\; \{0, 1, \ldots, \Omega_i\} \,\cup\, (\Omega_i, \infty). \tag{27}$$

At any time t, the compartment is said to be *jumping* if $V_i(t) \leq \Omega_i$ and *flowing* otherwise. Collecting indices, the state decomposes as $\mathbf{V} = (\mathbf{V}_J, \mathbf{V}_F)$, where $\mathbf{V}_J$ carries integer values and $\mathbf{V}_F$ real values. For notational simplicity we take a common threshold Ω below; the extension to species-specific Ω_i is immediate.

S.I.2.2 Reaction partition

Reactions are partitioned into a stochastic set $\mathcal{S}$ and a deterministic set $\mathcal{S}' = \mathcal{R} \setminus \mathcal{S}$:

$$\mathcal{S} \;=\; \big\{\mathcal{R}_k \;:\; \exists\, i \text{ with } V_i \in \mathbf{V}_J \text{ and } (\eta_{ik} \neq 0 \text{ or } \partial_{V_i}\lambda_k \neq 0)\big\}. \tag{28}$$

A reaction is thus treated stochastically if it either changes a jumping compartment or has a propensity that depends on one. After reordering indices, the stoichiometric matrix admits the block form

$$\eta \;=\; \left(\begin{array}{c|c} \eta_{\mathcal{S}'} & \bar{\eta}_{\mathcal{S}} \\ \hline 0 & \eta_{\mathcal{S}} \end{array}\right), \tag{29}$$

and between switching events the system obeys

$$\frac{\mathrm{d}\mathbf{V}_F}{\mathrm{d}t} = \eta_{\mathcal{S}'}\,\boldsymbol{\lambda}_{\mathcal{S}'}(\mathbf{V}) + \bar{\eta}_{\mathcal{S}}\,\boldsymbol{\Lambda}_{\mathcal{S}}(\mathbf{V}), \tag{30}$$

$$\mathbf{V}_J(t) = \mathbf{V}_J(t_0) + \eta_{\mathcal{S}} \int_{t_0}^{t} \boldsymbol{\Lambda}_{\mathcal{S}}(\mathbf{V}(s))\,\mathrm{d}s, \tag{31}$$

where $\boldsymbol{\Lambda}_{\mathcal{S}}$ is a stochastic vector of Dirac spike trains indexed by the firing times of reactions in $\mathcal{S}$. There are three event types: *jumps* (reactions in $\mathcal{S}$), *flows* (continuous evolution of $\mathbf{V}_F$ under $\mathcal{S}'$), and *switches* (threshold crossings that reclassify a compartment).

S.I.2.3 Jump-clock construction

Because $\mathbf{V}(t)$ varies continuously between jumps, the propensity $\lambda_k(\mathbf{V}(t))$ is time-dependent even in the absence of discrete events, and standard time-homogeneous Gillespie sampling is inapplicable. JSF samples the next firing time of each reaction $\mathcal{R}_k \in \mathcal{S}$ via a time-rescaling variant of the Next Reaction Method. The CDF of the next firing time, conditional on no firing since t_0, is

$$\mathrm{CDF}(t;k) \;=\; 1-\exp\Big(-\int_{t_0}^{t}\lambda_k(\mathbf{V}(s))\,\mathrm{d}s\Big). \tag{32}$$

Drawing $u_k \sim \mathrm{Unif}(0,1)$ and inverting gives the implicit equation $J_k(t_k)=0$ for the next firing time t_k, where

$$J_k(t) \;:=\; \log(u_k^{-1}) - \int_{t_0}^{t}\lambda_k(\mathbf{V}(s))\,\mathrm{d}s. \tag{33}$$

The quantity $J_k(t)$ is the *jump clock* for reaction $\mathcal{R}_k$: initialised at $\log(u_k^{-1})>0$, it decays monotonically, and a firing is triggered when it reaches zero.

S.I.2.4 Numerical integration of flows and clocks

Between jumps, $\mathbf{V}_J$ is constant and $\mathbf{V}_F$ evolves under the ODE portion of Equation (30). JSF integrates this forward in time with a fixed step Δt using an explicit scheme—forward Euler in the reference implementation [Germano et al., 2024], higher-order schemes being a drop-in replacement:

$$\mathbf{V}_F(t+\Delta t) \;=\; \mathbf{V}_F(t) + \Delta t\,\eta_{\mathcal{S}'}\,\boldsymbol{\lambda}_{\mathcal{S}'}(\mathbf{V}(t)). \tag{34}$$

Over the same step, each jump clock J_k is decremented by the integral of λ_k. Expanding $\lambda_k(\mathbf{V}(t+s))$ to first order in s yields

$$\Delta J_k \;=\; \int_0^{\Delta t}\lambda_k(\mathbf{V}(t+s))\,\mathrm{d}s \;\approx\; \frac{\Delta t}{2}\big(2\alpha+\beta\,\Delta t\big), \tag{35}$$

with $\alpha=\lambda_k(\mathbf{V}(t))$ and $\beta=\boldsymbol{\lambda}_{\mathcal{S}'}(\mathbf{V})^{\top}\eta_{\mathcal{S}'}^{\top}\nabla_{\mathbf{V}_F}\lambda_k$. If the provisional update $J_k(t)-\Delta J_k$ remains positive, no firing occurs during the step. If it becomes negative, the fractional time $\Delta\tau\in(0,\Delta t)$ at which the clock first reaches zero is obtained by solving the quadratic $2\Delta J_k=\Delta\tau\,(2\alpha+\beta\,\Delta\tau)$, giving

$$\Delta\tau \;=\; \begin{cases} \dfrac{\sqrt{\alpha^2+2\beta\,\Delta J_k}-\alpha}{\beta}, & \beta\neq 0,\\[2ex] \dfrac{\Delta J_k}{\alpha}, & \beta=0. \end{cases} \tag{36}$$

At $t+\Delta\tau$ the flow step is truncated, the stoichiometric update $\boldsymbol{\eta}_{\cdot k}$ is applied, and J_k is reinitialised with a fresh $u_k\sim\mathrm{Unif}(0,1)$.

S.I.2.5 Switching events

Switching events reclassify a compartment between jumping and flowing. Two cases arise.

Jumping to flowing. When a jump takes V_i from Ω to $\Omega+1$, compartment i is moved from $\mathbf{V}_J$ to $\mathbf{V}_F$. The continuous ODE for V_i is initialised at $\Omega+1$ and integrated thereafter as part of $\mathbf{V}_F$; $\mathcal{S}$ and $\mathcal{S}'$ are updated accordingly.

Flowing to jumping. When the flow takes V_i below Ω, compartment i is moved from $\mathbf{V}_F$ to $\mathbf{V}_J$. In general the crossing value $\hat{V}_i\in(\Omega-1,\Omega]$ is non-integer, so JSF applies a probabilistic rounding that preserves the conditional mean:

$$V_i \;=\; \begin{cases} \lceil\hat{V}_i\rceil & \text{with probability } \hat{V}_i-\lfloor\hat{V}_i\rfloor,\\ \lfloor\hat{V}_i\rfloor & \text{with probability } 1-(\hat{V}_i-\lfloor\hat{V}_i\rfloor). \end{cases} \tag{37}$$

This keeps $V_i\in\mathcal{V}_\Omega$ while ensuring $\mathbb{E}[V_i\mid\hat{V}_i]=\hat{V}_i$, preserving the first moment across the switch.

S.I.2.6 Algorithm

Algorithm 2 summarises one simulation step. The algorithm mirrors the reference implementation in the accompanying open-source package [Germano et al., 2024].

Algorithm 2 One JSF step (advance from t by at most Δt)

Input: state $\mathbf{V}$, partition (J, F), jump clocks $\{J_k\}_{k\in\mathcal{S}}$, step Δt
for $k \in \mathcal{S}$ **do** ▷ Provisional clock updates
  $\alpha_k \leftarrow \lambda_k(\mathbf{V})$; $\beta_k \leftarrow \boldsymbol{\lambda}_{\mathcal{S}'}(\mathbf{V})^\top \eta_{\mathcal{S}'}^\top \nabla_{\mathbf{V}_F} \lambda_k$
  $\Delta J_k \leftarrow \frac{\Delta t}{2}(2\alpha_k + \beta_k \Delta t)$
end for
$k^\star \leftarrow \arg\min_{k\in\mathcal{S},\, J_k-\Delta J_k<0} \Delta\tau_k$ ▷ Earliest firing, if any
if no k triggers (all $J_k - \Delta J_k \geq 0$) **then**
  $\mathbf{V}_F \leftarrow \mathbf{V}_F + \Delta t\, \eta_{\mathcal{S}'} \boldsymbol{\lambda}_{\mathcal{S}'}(\mathbf{V})$; $J_k \leftarrow J_k - \Delta J_k$ for all $k \in \mathcal{S}$
  $t \leftarrow t + \Delta t$
else
  $\Delta\tau \leftarrow \Delta\tau_{k^\star}$ ▷ From quadratic root above
  $\mathbf{V}_F \leftarrow \mathbf{V}_F + \Delta\tau\, \eta_{\mathcal{S}'} \boldsymbol{\lambda}_{\mathcal{S}'}(\mathbf{V})$ ▷ Truncated flow step
  Apply $\mathbf{V} \leftarrow \mathbf{V} + \boldsymbol{\eta}_{\cdot k^\star}$; reinitialise $J_{k^\star}$ with fresh $u \sim \mathrm{Unif}(0, 1)$
  $t \leftarrow t + \Delta\tau$
end if
for all i with V_i crossing Ω_i **do** ▷ Switching events
  Update partition (J, F); apply stochastic rounding if $F \to J$; update $\mathcal{S}, \mathcal{S}'$
end for
Output: updated $(\mathbf{V}, J, F, \{J_k\}, t)$

S.I.2.7 Relationship to the main-text formalism

The piecewise-deterministic generator $\mathcal{G}_t$ in Equation (6) is the infinitesimal description of the process implemented by Algorithm 2: the first summand encodes the jumps $\boldsymbol{\Lambda}_{\mathcal{S}}$ with propensities λ_k, and the gradient term encodes the flow $\eta_{\mathcal{S}'}\boldsymbol{\lambda}_{\mathcal{S}'}$. The partition $(J(t), F(t))$ in Equation (5) is determined by the current compartment values relative to Ω and is updated at switching events as described above. The error analysis in Appendix S.I.3 does not depend on the jump-clock construction or the numerical integration scheme; only on the fact that while $V_i \leq \Omega$ the dynamics agree with the CTMC, and while $V_i > \Omega$ they agree with the ODE.

S.I.3 Derivation of bound on extinction probability

In this section we provide a derivation of the bound on the extinction probability, $\Delta_\Omega(T)$, presented in the main text, Equation (18). We first bound the "late term" error, arising from up-crossings after the critical time s_* (see S.I.3.1). We then provide a detailed derivation for the "early term" error, which requires bounding stochastic excursions in the death-dominated regime using martingale inequalities (see S.I.3.2). In S.I.3.3 we combine these bounds and present the full algorithm. In S.I.3.4 we present and and discuss the simplified heuristic bound, cf. Equation (19).

S.I.3.1 Late term bound: birth-dominated regime

Consider first the late term. Up-crossings occurring after s_* represent the primary source of error because the birth-death balance favours persistence above the threshold. For the late term, we have:

$$\Delta_{\Omega,\text{late}}(T) \leq \mathbb{E}\left[[q_Y(\tau_\uparrow)]^{\Omega+1} \mathbf{1}\{s_* \leq \tau_\uparrow < T\}\right]. \tag{38}$$

While the process sits at Ω, the total birth intensity is $\Omega\lambda(t)$ (there are Ω individuals, each giving birth at rate $\lambda(t)$). The jump event $\Omega \to \Omega + 1$ is an inhomogeneous Poisson process, with density function

$$w(t) = \Omega\lambda(t) \exp\left(-\Omega \int_0^t \lambda(u) du\right). \tag{39}$$

At the up-crossing time $\tau_\uparrow = s$, the population is $Y(s) = \Omega + 1$, and by the branching property, the extinction probability is $[q_Y(s)]^{\Omega+1}$, giving:

$$\Delta_{\Omega,\text{late}}(T) \leq \int_{s_*}^{T} w(s)[q_Y(s)]^{\Omega+1}\, ds. \tag{40}$$

S.I.3.2 Early term bound: death-dominated regime

For the early term, the error arises from the difference in return times of excursions between the ODE and stochastic models on the window $[0, s_*]$. In this window, assume a uniform negative drift margin:

$$r(t) \leq -\rho < 0 \quad \text{for all } t \in [0, s_*], \qquad \rho := \inf_{t\in[0,s_*]} \big(-r(t)\big) > 0. \tag{41}$$

For an excursion that starts at time s (i.e., $Y(s^-) = \Omega$ and $Y(s) = \Omega + 1$), let $y^{(s)}$ denote the deterministic ODE trajectory solving $\dot{y}^{(s)}(t) = r(t)\, y^{(s)}(t)$ with $y^{(s)}(s) = \Omega + 1$, and let $\sigma(s)$ be its (deterministic) return time to level Ω, so $y^{(s)}(\sigma(s)) = \Omega$. We define the deviation process on this excursion by

$$M^{(s)}(t) := Y(t) - y^{(s)}(t), \qquad t \in [s, \sigma(s)]. \tag{42}$$

When s is clear we omit the superscript and write $M(t) = Y(t) - y(t)$; expressions such as $M(\sigma(s))$ are understood with this convention. Let $\tau_{\uparrow,k}$ be the k-th up-crossing time $\Omega \to \Omega{+}1$ (with $\tau_{\uparrow,k} < s_*$), let $\tau_{\downarrow,k}$ be the corresponding *stochastic* return time to Ω, and let $\sigma(\tau_{\uparrow,k})$ be the *deterministic* return time obtained by evolving the ODE segment from $y(\tau_{\uparrow,k}) = \Omega{+}1$ until it hits Ω:

$$\int_{\tau_{\uparrow,k}}^{\sigma(\tau_{\uparrow,k})} r(u)\, du = \log\!\left(\frac{\Omega}{\Omega+1}\right). \tag{43}$$

Write

$$p(t) := \big[q_Y(t)\big]^{\Omega} \tag{44}$$

for the extinction probability at horizon T when starting from Ω individuals at time t; $p(t)$ is decreasing on $[0, s_*]$ and L_{eff}-Lipschitz there, where

$$L_{\text{eff}} := \sup_{s\in[0,s_*]} |p'(s)|, \qquad p'(s) = \Omega\, q_Y(s)^{\Omega-1} \left(\mu(s)\,(q_Y(s) - 1) + \lambda(s)\,(q_Y(s) - q_Y(s)^2)\right). \tag{45}$$

The per-excursion contribution to the error is

$$\theta(\tau_{\uparrow,k}) := \max\{0, p\big(\sigma(\tau_{\uparrow,k})\big) - p\big(\tau_{\downarrow,k}\big)\} \tag{46}$$

(taking the positive part so "helpful" negative excursions do not cancel "harmful" ones). Summing over all early excursions,

$$\Delta_{\Omega,\text{early}}(T) = \mathbb{E}\left[\sum_{k=1}^{N_\uparrow} \theta(\tau_{\uparrow,k})\right] \leq \mathbb{E}[N_\uparrow] \sup_{s\in[0,s_*]} m(s) \tag{47}$$

where $N_\uparrow$ is the number of up-crossings with start time $< s_*$ and

$$m(s) \ := \ \mathbb{E}[\theta(s) \,|\, \tau_{\uparrow,1} = s] \tag{48}$$

is the expected per-excursion contribution when an excursion starts at time s.

Let $N_\uparrow(t)$ count up-crossings $\Omega \to \Omega{+}1$ up to time t. While $Y(t) = \Omega$, the instantaneous intensity of up-crossings is $\Omega\,\lambda(t)$, so the compensator of $N_\uparrow$ on $[0, s_*]$ is $\int_0^{s_*} \Omega\,\lambda(t)\,\mathbf{1}_{\{Y(t)=\Omega\}}\, dt$. Taking expectations yields

$$\mathbb{E}[N_\uparrow] = \mathbb{E}\bigg[\int_0^{s_*} \Omega\,\lambda(t)\,\mathbf{1}_{\{Y(t)=\Omega\}}\, dt\bigg] \ \leq \ \int_0^{s_*} \Omega\,\lambda(t)\, dt, \tag{49}$$

and, if desired, the crude simplification $\mathbb{E}[N_\uparrow] \leq \Omega\,\bar{\lambda}\, s_*$ with $\bar{\lambda} := \sup_{t\in[0,s_*]} \lambda(t)$. To complete the bound, we must estimate $m(s)$, the expected per-excursion error contribution. The argument proceeds in three main steps: first, we use the smoothness of the extinction probability $p(t)$ to relate the error to the excursion's time duration; second, we bound this duration using a drift argument; and third, we bound the stochastic fluctuations using a martingale inequality.

Since $p(t)$ is L_{eff}-Lipschitz and decreasing on $[0, s_*]$, we can bound the error contribution $\theta(s)$ by the difference in return times:

$$\theta(s) \leq L_{\text{eff}} \max\{0, \tau_\downarrow - \sigma(s)\}. \tag{50}$$

The problem thus reduces to finding the expected time the stochastic process $Y(t)$ takes to return to Ω after the deterministic trajectory $y(t)$ has already done so at time $\sigma(s)$.

At $t = \sigma(s)$, the deterministic trajectory sits at level Ω, i.e. $y(\sigma(s)) = \Omega$. Any excess population in the stochastic process is given by $\phi(s) := \max\{0,\, Y(\sigma(s)) - \Omega\} = \max\{0,\, M(\sigma(s))\} \leq \sup_{t\in[s,\sigma(s)]} |M(t)|$. For the process to return to Ω, this excess must be depleted. During this return interval, $[\sigma(s), \tau_\downarrow)$, the population size $Y(t)$ is at least Ω and the per-capita growth rate $r(t)$ is at most $-\rho$. Consequently, the total population drift $r(t)Y(t)$ is bounded above by $-\rho\Omega$ [3]. Applying the Optional Stopping Theorem to the process from time $\sigma(s)$ until the return time $\tau_\downarrow$ provides a bound on the expected time to drain this excess:

$$\mathbb{E}\left[\tau_\downarrow - \sigma(s) \mid \mathcal{F}_{\sigma(s)}\right] \leq \frac{\phi(s)}{\rho\Omega}. \tag{51}$$

Taking expectations conditional on the excursion starting at s and combining with the Lipschitz bound yields:

$$m(s) = \mathbb{E}[\theta(s) \mid \tau_{\uparrow,1} = s] \leq \frac{L_{\text{eff}}}{\rho\Omega}\mathbb{E}[\phi(s) \mid \tau_{\uparrow,1} = s]. \tag{52}$$

The excess population $\phi(s)$ is itself bounded by the maximum deviation between the stochastic and deterministic paths, $M(t) := Y(t) - y(t)$, over the excursion interval. This leads to:

$$m(s) \leq \frac{L_{\text{eff}}}{\rho\Omega}\mathbb{E}\left[\sup_{t\in[s,\sigma(s)]} |M(t)| \middle| \tau_{\uparrow,1} = s\right]. \tag{53}$$

The remainder of this subsection is dedicated to bounding the expected supremum $\mathbb{E}[\sup|M(t)|]$ using martingale analysis. The argument proceeds as follows:

- First, we use a martingale decomposition and Grönwall's inequality to relate the supremum of the full deviation, $\sup|M(t)|$, to the supremum of its core martingale component.
- Second, we apply the Burkholder-Davis-Gundy (BDG) inequality to bound the martingale supremum in terms of its predictable quadratic variation (PQV).
- Third, we derive a uniform, deterministic bound $V_\star$ for this PQV.
- Finally, we assemble these components to complete the early term bound.

S.I.3.2.1 Martingale Decomposition and Grönwall's Inequality

Throughout this analysis, we analyse a single excursion that starts at time s (an up-crossing $Y(s^-) = \Omega$, $Y(s) = \Omega{+}1$). All martingales are taken with respect to the shifted filtration $(\mathcal{F}_{s+t})_{t\geq 0}$, and all expectations may be read conditionally on $\mathcal{F}_s$ (equivalently, on the event $\{\tau_{\uparrow,1} = s\}$ when we condition on the value of the first up-crossing time). This conditioning does not change any inequality below: the Grönwall step is pathwise, and martingale inequalities such as BDG and Freedman hold with the same universal constants under the conditional law.

Define the compensated càdlàg martingale increment on $[s,\infty)$:

$$\tilde{M}^{(s)}(t) \;:=\; \tilde{M}(t) - \tilde{M}(s) \;=\; Y(t) - Y(s) - \int_s^t r(u)\,Y(u)\,du, \qquad t \geq s, \tag{54}$$

so that $|\Delta\tilde{M}^{(s)}| \leq 1$ and the predictable quadratic variation is

$$\left\langle \tilde{M}^{(s)} \right\rangle_{[s,t]} \;=\; \int_s^t \big(\lambda(u) + \mu(u)\big)\,Y(u)\,du. \tag{55}$$

Let $y^{(s)}$ solve $\dot{y}^{(s)}(t) = r(t)\,y^{(s)}(t)$ with $y^{(s)}(s) = \Omega{+}1$, and set $M(t) := Y(t) - y^{(s)}(t)$. Since excursions start at $Y(s) = y^{(s)}(s) = \Omega{+}1$, we have $M(s) = 0$, and

$$M(t) \;=\; \tilde{M}^{(s)}(t) \;+\; \int_s^t r(u)\,M(u)\,du. \tag{56}$$

[3] Think of a tank that contains $\phi(s)$ extra units and drains at a *minimum* rate $\rho\,\Omega$ whenever it is above Ω: at such a rate, the time to remove $\phi(s)$ units cannot exceed $\phi(s)/(\rho\,\Omega)$ on average.

Define the envelopes $\phi(t) := \sup_{u\in[s,t]} |M(u)|$ and $\psi(t) := \sup_{u\in[s,t]} |\tilde{M}^{(s)}(u)|$. Then

$$\phi(t) \;\le\; \psi(t) + \int_s^t |r(v)|\,\phi(v)\,dv, \tag{57}$$

and by Grönwall's inequality [Grönwall, 1919, Walter, 1998], for any $t \in [s, \sigma(s)]$,

$$\sup_{u\in[s,t]} |M(u)| \;\le\; \exp\Big(\int_s^t |r(v)|\,dv\Big) \sup_{u\in[s,t]} |\tilde{M}^{(s)}(u)|. \tag{58}$$

On the early window $r \le -\rho < 0$ and using $\int_s^{\sigma(s)} r(u)\,du = \log\big(\Omega/(\Omega{+}1)\big)$, we obtain

$$\sup_{u\le\sigma(s)} |M(u)| \;\le\; \frac{\Omega+1}{\Omega} \sup_{u\le\sigma(s)} |\tilde{M}^{(s)}(u)|. \tag{59}$$

Taking *conditional* expectations (given $\mathcal{F}_s$) gives

$$\mathbb{E}\left[\sup_{t\le\sigma(s)} |M(t)| \,\middle|\, \mathcal{F}_s\right] \;\le\; \frac{\Omega+1}{\Omega}\,\mathbb{E}\left[\sup_{t\le\sigma(s)} |\tilde{M}^{(s)}(t)| \,\middle|\, \mathcal{F}_s\right]. \tag{60}$$

S.I.3.2.2 Bounding the Martingale supremum via BDG

Freedman's inequality yields for all $x > 0$,

$$\Pr\left(\sup_{t\le\sigma(s)} \left|\tilde{M}^{(s)}(t)\right| \ge x \,\middle|\, \mathcal{F}_s\right) \;\le\; 2\exp\left(-\frac{x^2}{2\big(V + x/3\big)}\right), \tag{61}$$

whenever $\langle \tilde{M}^{(s)}\rangle_{[s,\sigma(s)]} \le V$ almost surely (bounded jumps ≤ 1). Consequently,

$$\mathbb{E}\left[\sup_{t\le\sigma(s)} \left|\tilde{M}^{(s)}(t)\right| \,\middle|\, \mathcal{F}_s\right] \;\le\; \int_0^\infty 2\exp\left(-\frac{x^2}{2(V + x/3)}\right) dx. \tag{62}$$

Rather than handling the long tail numerically, one can conveniently bypass it with the Burkholder–Davis–Gundy (BDG) inequality [Burkholder, 1966, Davis, 1970, Burkholder and Gundy, 1970] . Taking the 1st moment ($p = 1$) yields a universal constant C_{BDG} (we take $C_{\mathrm{BDG}} = 2$) such that

$$\mathbb{E}\left[\sup_{t\le\sigma(s)} \left|\tilde{M}^{(s)}(t)\right| \,\middle|\, \mathcal{F}_s\right] \;\le\; C_{\mathrm{BDG}}\,\mathbb{E}\left[\langle \tilde{M}^{(s)}\rangle^{1/2}_{[s,\sigma(s)]} \,\middle|\, \mathcal{F}_s\right]. \tag{63}$$

If, in addition, we have the uniform bound (see Appendix S.I.3.2.3)

$$\mathbb{E}\left[\langle \tilde{M}^{(s)}\rangle_{[s,\sigma(s)]} \,\middle|\, \mathcal{F}_s\right] \;\le\; V_\star \qquad \text{for all } s \in [0, s_*], \tag{64}$$

then Jensen's inequality (concavity of $\sqrt{\cdot}$) yields

$$\mathbb{E}\left[\sup_{t\le\sigma(s)} \left|\tilde{M}^{(s)}(t)\right| \,\middle|\, \mathcal{F}_s\right] \;\le\; C_{\mathrm{BDG}}\,\sqrt{V_\star}. \tag{65}$$

Combining Equations (60) and (65),

$$\mathbb{E}\left[\sup_{t\le\sigma(s)} |M(t)| \,\middle|\, \mathcal{F}_s\right] \;\le\; \frac{\Omega+1}{\Omega}\,C_{\mathrm{BDG}}\,\sqrt{V_\star}. \tag{66}$$

uniformly in $s \in [0, s_*]$. This is the estimate invoked in the early-term bound in the main text.

S.I.3.2.3 Bounding the predictable quadratic variation

This section is concerned with calculating $V_\star$, a deterministic bound on the conditional expected predictable quadratic variation (PQV) accumulated over any early excursion. $V_\star$ plays a crucial role in controlling the early excursion behaviour of the birth–death process. The calculation of $V_\star$ allows us to establish concrete bounds on the martingale deviation through the application of the BDG inequality, cf. Equation (73)

In the general time-inhomogeneous birth–death case, on an excursion started at time s with $Y(s) = \Omega + 1$, the compensated jump martingale $\tilde{M}^{(s)}(t) := Y(t) - Y(s) - \int_s^t r(u)Y(u)\,du$ has PQV

$$\langle \tilde{M}^{(s)} \rangle_{[s,t]} = \int_s^t \big(\lambda(u) + \mu(u)\big)\, Y(u)\, du, \qquad r(u) = \lambda(u) - \mu(u). \tag{67}$$

For $t \in [s, \sigma(s)]$,

$$\mathbb{E}\Big[\langle \tilde{M}^{(s)} \rangle_{[s,\sigma(s)]} \,\Big|\, \mathcal{F}_s\Big] = \int_s^{\sigma(s)} \big(\lambda(u) + \mu(u)\big)\, \mathbb{E}[Y(u) \,|\, \mathcal{F}_s]\, du. \tag{68}$$

Since the per-capita rates depend only on time, $\mathbb{E}[Y(u) \mid \mathcal{F}_s]$ solves the same linear ODE as the deterministic envelope $y^{(s)}$: $\frac{d}{du}\, y^{(s)}(u) = r(u)\, y^{(s)}(u),\ y^{(s)}(s) = \Omega + 1$, hence

$$\mathbb{E}\Big[\langle \tilde{M}^{(s)} \rangle_{[s,\sigma(s)]} \,\Big|\, \mathcal{F}_s\Big] = \int_s^{\sigma(s)} \big(\lambda(u) + \mu(u)\big)\, y^{(s)}(u)\, du \ \le\ V_\star, \tag{69}$$

with

$$V_\star \ := \sup_{s \in [0, s_*]} \int_s^{\sigma(s)} \big(\lambda(u) + \mu(u)\big)\, y^{(s)}(u)\, du. \tag{70}$$

Using the early-window negativity $r \le -\rho < 0$ and $y^{(s)}(u) \le (\Omega + 1)e^{-\rho(u-s)}$, we get the convenient crude bound

$$V_\star \ \le \sup_{t \in [0, s_*]} \big(\lambda(t) + \mu(t)\big)\, (\Omega + 1) \int_0^\infty e^{-\rho w}\, dw \ = \ \frac{\Omega + 1}{\rho} \sup_{t \in [0, s_*]} \big(\lambda(t) + \mu(t)\big). \tag{71}$$

S.I.3.2.4 Completing the early term bound

We now have all the pieces to bound the per-excursion error $m(s)$. Substituting the martingale bound from Equation (66) into our expression for $m(s)$ in Equation (53) yields a uniform bound on the per-excursion error:

$$\sup_{s \in [0, s_*]} m(s) \le \frac{L_{\text{eff}}}{\rho \Omega} \left(\frac{\Omega + 1}{\Omega}\, C_{\text{BDG}}\, \sqrt{V_\star} \right). \tag{72}$$

Finally, substituting this bound for $m(s)$ into Equation (47) along with the estimate for $\mathbb{E}[N_\uparrow]$ from Equation (49) yields the final estimate for the total early-term error:

$$\Delta_{\Omega,\text{early}}(T) \le \left(\int_0^{s_*} \Omega\, \lambda(t)\, dt \right) \frac{L_{\text{eff}}}{\rho} \frac{\Omega + 1}{\Omega} C_{\text{BDG}} \sqrt{V_\star}. \tag{73}$$

S.I.3.3 Final combined bound and algorithm

Putting together Equations (73) and (40) yields

$$\Delta_\Omega(T) \le \left(\int_0^{s_*} \Omega \lambda(t)\, dt \right) \frac{L_{\text{eff}}}{\rho} \frac{\Omega + 1}{\Omega} C_{\text{BDG}} \sqrt{V_\star} + \int_{s_*}^T \Omega \lambda(s) e^{-\Omega \int_0^s \lambda} [q(s)]^{\Omega + 1}\, ds. \tag{74}$$

Algorithm 3 outlines the complete procedure for computing the formal bound.

Algorithm 3 Full Error Bound for Birth-Death Systems

Input: Threshold Ω, time horizon T, birth rate $\lambda(t)$, death rate $\mu(t)$

▷ *Part 1: Preliminary Calculations*

Compute net growth rate: $r(t) = \lambda(t) - \mu(t)$

Find critical time: $t_c = \inf\{t \geq 0 : r(t) \geq 0\}$

Solve for point of no return s_* from: $\int_{s_*}^{t_c} r(u)\,du = \log\left(\frac{\Omega}{\Omega+1}\right)$

Solve Riccati equation backward for extinction probability $q_Y(s)$ on $[0, T]$:
$\frac{dq_Y}{ds} = \mu(s)(q_Y - 1) + \lambda(s)(q_Y - q_Y^2)$, with terminal condition $q_Y(T) = 0$

▷ *Part 2: Early Excursion Error Term on* $[0, s_*]$

Compute negative drift margin: $\rho = \inf_{t\in[0,s_*]}(-r(t))$

Compute expected number of up-crossings: $\mathbb{E}[N_\uparrow] = \int_0^{s_*} \Omega\lambda(t)\,dt$

Define extinction probability from Ω: $p(t) = [q_Y(t)]^\Omega$

Compute effective Lipschitz constant: $L_{\text{eff}} = \sup_{s\in[0,s_*]} |p'(s)|$

Compute an upper bound $V_\star$ for the martingale quadratic variation on $[0, s_*]$

Assemble early term bound (where C_{BDG} is the Burkholder-Davis-Gundy constant):
$\Delta_{\Omega,\text{early}}(T) = \mathbb{E}[N_\uparrow] \cdot \frac{L_{\text{eff}}}{\rho}\frac{\Omega+1}{\Omega} C_{\text{BDG}}\sqrt{V_\star}$

▷ *Part 3: Late Excursion Error Term on* $[s_*, T]$

Compute the up-crossing time density: $w(s) = \Omega\lambda(s)\exp\left(-\Omega\int_0^s \lambda(u)\,du\right)$

Compute the late term bound via integration:
$\Delta_{\Omega,\text{late}}(T) = \int_{s_*}^T w(s)[q_Y(s)]^{\Omega+1}\,ds$

▷ *Part 4: Final Bound*

Combine the two terms: $\Delta_\Omega(T) = \Delta_{\Omega,\text{early}}(T) + \Delta_{\Omega,\text{late}}(T)$

Output: Full error bound $\Delta_\Omega(T)$

S.I.3.4 Heuristic late-only bound

As a fast and implementable approximation, we retain only the *late* contribution (up-crossings after s_*) and neglect the early term. This is often appropriate in regimes where negative drift on $[0, s_*]$ quickly corrects early excursions, so most discrepancy arises when a path up-crosses and fails to return before the drift turns nonnegative.

The key practical insight is that in most birth-death systems, the late term dominates the error budget. When the death rate significantly exceeds the birth rate during the early period ($r(t) \ll 0$ for $t \in [0, s_*]$), excursions above the threshold are rapidly corrected, making the early term contribution negligible.

Starting from the general late bound with the nonhomogeneous up-crossing density $w(s) = \Omega\,\lambda(s)\exp\{-\Omega\int_0^s \lambda(u)\,du\}$, we have:

$$\Delta_{\Omega,\text{late}}(T) \leq \int_{s_*}^{T} \Omega\,\lambda(s)\,e^{-\Omega\int_0^s \lambda(u)\,du}\,[q_Y(s)]^{\Omega+1}\,ds. \tag{75}$$

We can obtain a simple analytic upper bound by replacing λ with $\bar{\lambda} := \sup_{[0,T]} \lambda$ and using that $q_Y(\cdot)$ is decreasing:

$$\Delta_{\Omega,\text{late}}(T) \leq \int_{s_*}^{T} \Omega\,\bar{\lambda}\,e^{-\Omega\bar{\lambda}s}\,[q_Y(s)]^{\Omega+1}\,ds \leq [q_Y(s_*)]^{\Omega+1}. \tag{76}$$

If a more conservative bound is preferred, we can replace the exponent $\Omega + 1$ with Ω:

$$\Delta_\Omega(T) \lesssim [q_Y(s_*)]^\Omega. \tag{77}$$

This bound is looser but may be more convenient for reporting single-number accuracy guarantees.

S.I.4 Calculating s_* (Lambert-W)

We want to find the value $s_* \in [0, t_c]$ that satisfies the integral criterion:

$$\int_{s_*}^{t_c} r(u)\,du = \log\left(\frac{\Omega}{\Omega+1}\right) \tag{78}$$

where the rate function is $r(t) = \alpha - \beta x_2(0)e^{-\gamma t}$. The upper limit of integration, t_c, is the time at which $r(t_c) = 0$, given by $t_c = \frac{1}{\gamma}\log(\frac{B}{\alpha})$, assuming $B := \beta x_2(0) > \alpha$.

S.I.4.1 Numerical solution

We can solve for s_* by finding the root of a one-dimensional, monotone equation. Let's define a function $F(s)$ on the interval $[0, t_c]$ such that s_* is its root. First, let $L := \log(\frac{\Omega}{\Omega+1})$, which is a negative constant. The integral can be calculated explicitly:

$$I(s) := \int_s^{t_c} r(u)\,du = \left[\alpha u + \frac{B}{\gamma}e^{-\gamma u}\right]_s^{t_c} = \alpha(t_c - s) + \frac{B}{\gamma}\left(e^{-\gamma t_c} - e^{-\gamma s}\right) \tag{79}$$

Using the fact that $e^{-\gamma t_c} = \alpha/B$, this simplifies to:

$$I(s) = \alpha(t_c - s) + \frac{\alpha}{\gamma} - \frac{B}{\gamma}e^{-\gamma s} \tag{80}$$

The value s_* is the solution to $I(s_*) = L$. We can define the function to solve as:

$$F(s) := I(s) - L = 0 \tag{81}$$

This function is continuous and strictly increasing on $[0, t_c]$ because its derivative is positive for $s < t_c$:

$$F'(s) = -r(s) = -(\alpha - Be^{-\gamma s}) = Be^{-\gamma s} - \alpha > 0 \tag{82}$$

The values at the interval's endpoints are $F(t_c) = I(t_c) - L = 0 - L = -L > 0$ and $F(0) = \alpha t_c + \frac{\alpha}{\gamma} - \frac{B}{\gamma} - L$. Since $F(s)$ is monotone and $F(t_c) > 0$, a unique root $s_* \in [0, t_c)$ exists if and only if $F(0) \leq 0$. If $F(0) > 0$, no such root exists, and we take $s_* := 0$. Given a bracket $[a, b] = [0, t_c]$ where $F(a) \leq 0 < F(b)$, the unique root can be found efficiently using (e.g.) bisection method.

S.I.4.2 Closed-form solution

Alternatively, the equation can be solved analytically to yield a closed-form expression for s_*. Starting from the integrated equation $I(s_*) = L$:

$$\alpha(t_c - s_*) + \frac{\alpha}{\gamma} - \frac{B}{\gamma}e^{-\gamma s_*} = L \tag{83}$$

We rearrange and substitute $t_c = \frac{1}{\gamma}\log(\frac{B}{\alpha})$:

$$\frac{\alpha}{\gamma}\log\left(\frac{B}{\alpha}\right) + \frac{\alpha}{\gamma} - L = \alpha s_* + \frac{B}{\gamma}e^{-\gamma s_*} \tag{84}$$

Multiplying by $\frac{\gamma}{\alpha}$ gives:

$$\log\left(\frac{B}{\alpha}\right) + 1 - \frac{\gamma L}{\alpha} = \gamma s_* + \frac{B}{\alpha}e^{-\gamma s_*} \tag{85}$$

Let's introduce a change of variables with $u := \frac{B}{\alpha}e^{-\gamma s_*}$. This implies $\gamma s_* = \log(\frac{B}{\alpha u}) = \log(\frac{B}{\alpha}) - \log(u)$. Substituting these into the equation yields:

$$\log\left(\frac{B}{\alpha}\right) + 1 - \frac{\gamma L}{\alpha} = \left(\log\left(\frac{B}{\alpha}\right) - \log u\right) + u \tag{86}$$

The $\log(\frac{B}{\alpha})$ terms cancel, leaving the transcendental equation:

$$u - \log u = C, \qquad \text{where} \qquad C := 1 - \frac{\gamma L}{\alpha} \tag{87}$$

This equation can be solved using the Lambert-W function. By rearranging, we get $ue^{-u} = e^{-C}$. The solution for u is $u = -W(-e^{-C})$. Since $L < 0$, we have $C > 1$, which means there are two possible real solutions, corresponding to the W_0 and W_{-1} branches of the function. The physically relevant solution must satisfy $s_* \in [0, t_c)$, which corresponds to $u \in [1, B/\alpha]$. This requires the W_{-1} branch.

Solving for s_* from $u = \frac{B}{\alpha}e^{-\gamma s_*}$ gives the final closed-form expression:

$$s_* = -\frac{1}{\gamma}\log\left(\frac{\alpha}{B}\left[-W_{-1}\left(-e^{-C}\right)\right]\right) \in [0, t_c) \tag{88}$$

S.I.5 Lotka–Volterra model: Detailed derivations

This appendix provides explicit formulas for the Lotka–Volterra predator–prey system introduced in Section 5.1. Recall the rates from Equation (24):

$$\dot{x}_2(t) = -\gamma x_2(t), \qquad x_2(t) = x_{2,0}e^{-\gamma t}, \qquad \lambda(t) \equiv \alpha, \quad \mu(t) = \beta x_2(t), \quad r(t) = \alpha - \beta x_2(t), \tag{24}$$

where $\alpha, \beta, \gamma > 0$ are fixed parameters, the prey $Y = X_1$ is the focal species, and the predator X_2 evolves deterministically.

S.I.5.1 Derivation of the LV bound

The sign change of $r(t) = \alpha - \beta x_{2,0}e^{-\gamma t}$ determines the critical time t_c from $r(t_c) = 0$:

$$t_c = \frac{1}{\gamma}\log\left(\frac{\beta x_{2,0}}{\alpha}\right) \quad \text{if } \beta x_{2,0} > \alpha, \quad \text{else } t_c = 0. \tag{89}$$

The point of no return $s_\star \in [0, t_c)$ is defined by

$$\int_{s_\star}^{t_c} \left(\alpha - \beta x_{2,0}e^{-\gamma u}\right) du = \log\left(\frac{\Omega}{\Omega+1}\right), \tag{90}$$

with a closed form via the Lambert W function given in Appendix S.I.4.

With $\lambda \equiv \alpha$ and $\mu = \beta x_2$, the backward Kolmogorov equation for the one-lineage extinction probability is

$$\frac{dq_Y}{ds} = \mu(s)(q_Y - 1) + \lambda(s)(q_Y - q_Y^2) = \beta x_{2,0}e^{-\gamma s}(q_Y - 1) + \alpha(q_Y - q_Y^2), \quad q_Y(T) = 0. \tag{91}$$

For initial condition $Y(0) = \Omega$, the exact extinction probability by horizon T is $P_{\text{ext}}(T) = [q_Y(0)]^\Omega$.

The up-crossing intensity on $[s_\star, T]$ yields

$$\Delta_{\Omega,\text{late}}(T) \le \int_{s_\star}^{T} \Omega\,\lambda(s)\,[q_Y(s)]^{\Omega+1}\,ds = \int_{s_\star}^{T} \Omega\,\alpha\,[q_Y(s)]^{\Omega+1}\,ds. \tag{92}$$

A one-number summary follows from the monotonicity of q_Y:

$$\Delta_{\Omega,\text{late}}(T) \le [q_Y(s_\star)]^{\Omega+1}\,\min\{1, \Omega\alpha(T - s_\star)\} \le [q_Y(s_\star)]^{\Omega+1}. \tag{93}$$

On $[0, s_\star]$, the growth rate remains strictly negative. Define the uniform negative margin

$$\rho := \inf_{t\in[0,s_\star]}(\mu(t) - \lambda(t)) = \inf_{t\in[0,s_\star]}(\beta x_{2,0}e^{-\gamma t} - \alpha) = \beta x_{2,0}e^{-\gamma s_\star} - \alpha > 0. \tag{94}$$

Then $\mathbb{E}[N_\uparrow] \le \int_0^{s_\star} \Omega\lambda(t)\,dt = \Omega\alpha s_\star$, and the LV version of the early bound is

$$\Delta_{\Omega,\text{early}}(T) \le \underbrace{\Omega\,\alpha\,s_\star}_{\mathbb{E}[N_\uparrow]}\,\frac{L_{\text{eff}}}{\rho}\,\frac{\Omega+1}{\Omega}\,C_{\text{BDG}}\,\sqrt{V_\star}, \tag{95}$$

with a convenient deterministic bound (see Appendix S.I.5.2)

$$V_\star \le \frac{\Omega+1}{\rho}\sup_{t\in[0,s_\star]}\big(\lambda(t) + \mu(t)\big) = \frac{\Omega+1}{\rho}\sup_{t\in[0,s_\star]}\left(\alpha + \beta x_{2,0}e^{-\gamma t}\right) = \frac{\Omega+1}{\rho}\big(\alpha + \beta x_{2,0}\big). \tag{96}$$

Adding Equations (92) and (95) gives the Lotka–Volterra instance of the general bound:

$$\Delta_\Omega(T) \le \Big(\Omega\alpha s_\star\Big)\,\frac{L_{\text{eff}}}{\rho}\,\frac{\Omega+1}{\Omega}\,C_{\text{BDG}}\sqrt{V_\star} + \int_{s_\star}^{T} \Omega\alpha\,[q_Y(s)]^{\Omega+1}\,ds. \tag{97}$$

S.I.5.2 Variance term $V_\star$: exact and approximate formulas

For completeness, we derive the predictable quadratic variation bound $V_\star$ specialised to the deterministic predator case. With $r(t) = \alpha - \beta x_2(t)$ and $x_2(t) = x_{2,0}e^{-\gamma t}$, the exact expression is

$$V_\star = \sup_{s\in[0,s_\star]} \int_s^{\sigma(s)} \big(\alpha + \beta x_2(u)\big)\, y^{(s)}(u)\, du. \tag{98}$$

There are two useful forms:

1. **Option A (simple bound).**
   Using $y^{(s)}(u) \le (\Omega+1)e^{-\rho(u-s)}$ and $\sup_{[0,s_\star]}(\alpha+\beta x_2) < \infty$,
   $$V_\star \le \frac{\Omega+1}{\rho} \sup_{t\in[0,s_\star]} \big(\alpha + \beta x_2(t)\big). \tag{99}$$

2. **Option B (tighter expression).** Let
   $$R(t) = \int_0^t r(u)\, du, \quad G(t) = (\alpha + \beta x_2(t))e^{R(t)}, \quad H(t) = \int_0^t G(u)\, du. \tag{100}$$
   Then $y^{(s)}(u) = (\Omega+1)e^{R(u)-R(s)}$ and $R(\sigma(s)) - R(s) = \log\big(\Omega/(\Omega+1)\big)$, so
   $$\int_s^{\sigma(s)} \big(\alpha + \beta x_2(u)\big)\, y^{(s)}(u)\, du = (\Omega+1)e^{-R(s)}\big(H(\sigma(s)) - H(s)\big), \tag{101}$$
   hence
   $$V_\star = \sup_{s\in[0,s_\star]} (\Omega+1)\, e^{-R(s)}\big(H(\sigma(s)) - H(s)\big). \tag{102}$$
